# Development and validation of a new stage-specific nomogram model for predicting cancer-specific survival in patients in different stages of colon cancer: A SEER population-based study and external validation

**DOI:** 10.3389/fonc.2022.1024467

**Published:** 2022-12-07

**Authors:** Chenhao Hu, Feiyu Shi, Zhe Zhang, Lei Zhang, Ruihan Liu, Xuejun Sun, Liansheng Zheng, Junjun She

**Affiliations:** ^1^ Department of General Surgery, The First Affiliated Hospital of Xi’an Jiaotong University, Xi’an, Shaanxi, China; ^2^ Center for Gut Microbiome Research, Med-X Institute, The First Affiliated Hospital of Xi’an Jiaotong University, Xi’an, Shaanxi, China; ^3^ Department of High Talent, The First Affiliated Hospital of Xi’an Jiaotong University, Xi’an, Shaanxi, China; ^4^ Department of Digestive Minimally Invasive Surgery, The Second Affiliated Hospital of Baotou Medical College, Baotou, China

**Keywords:** colorectal cancer, SEER, risk model, nomogram, survival

## Abstract

**Background:**

The effects of laterality of the primary tumor on survival in patients in different stages of colon cancer are contradictory. We still lack a strictly evaluated and validated survival prediction tool, considering the different roles of tumor laterality in different stages.

**Methods:**

A total of 101,277 and 809 colon cancer cases were reviewed using the Surveillance, Epidemiology, and End Results database and the First Affiliated Hospital of Xi ‘an Jiaotong University database, respectively. We established training sets, internal validation sets and external validation sets. We developed and evaluated stage-specific prediction models and unified prediction models to predict cancer-specific survival and compared the prediction abilities of these models.

**Results:**

Compared with right-sided colon cancers, the risk of cancer-specific death of left-sided colon cancer patients was significantly higher in stage I/II but was markedly lower in stage III patients. We established stage-specific prediction models for stage I/II and stage III separately and established a unified prediction model for all stages. By evaluating and validating the validation sets, we reported high prediction ability and generalizability of the models. Furthermore, the stage-specific prediction models had better predictive power and efficiency than the unified model.

**Conclusions:**

Right-sided colon cancer patients have better cancer-specific survival than left-sided colon cancer patients in stage I/II and worse cancer-specific survival in stage III. Using stage-specific prediction models can further improve the prediction of cancer-specific survival in colon cancer patients and guide clinical decisions.

## Introduction

Colon cancer remains one of the most commonly diagnosed malignancy and leading cause of cancer-related deaths worldwide, and the morbidity and mortality rate of colon cancer has been increasing in recent years (1). In recent decades, increasing evidence has suggested that the laterality of the primary tumor is an effective prognostic factor for colon cancer. A meta-analysis of 66 relevant studies involving 1,437,846 patients suggested that the risk of death was significantly reduced in patients with left-sided primary tumors and demonstrated that the laterality of the primary tumor should be considered when deciding the ideal treatment method (2). In addition, several studies have shown that left-sided and right-sided colon cancers harbor different clinical, pathobiologic and molecular characteristics (3, 4). Moreover, the laterality of the primary tumor may be associated with the response to adjuvant therapy and targeted therapy and has an underlying predictive power for evaluating the survival benefit of targeted therapy (4, 5).

However, several recent studies provide new evidence suggesting that the relationship between survival and the laterality of the primary tumor is stage dependent. Compared with left-sided colon cancer, in stage II, the risk of mortality was significantly lower in right-sided colon cancer patients, and was markedly higher in right-sided colon cancer patients in stage III (6–9). Due to the different contributions of laterality to the prognosis of colon cancer in stage II and stage III, using a unified prediction model to predict survival in cancer patients inevitably leads to the misestimation of survival in some patients. However, to date, stage-specific prediction models based on large populations are still limited. Given that a powerful prognostic prediction tool plays a crucial role in deciding the appropriate therapy to improve survival, it is necessary to discuss and develop stage-specific prediction models for stage II and stage III separately to increase the accuracy of survival prediction.

Therefore, in this study, we retrieved and extracted data from the Surveillance, Epidemiology, and End Results (SEER) incidence database and the Electronic Medical Record and Analysis System (EMRAS) of the First Affiliated Hospital of Xi ‘an Jiaotong University. We developed prediction models based on the training data set of SEER data to predict cancer-specific survival, and evaluated and validated models using internal and external validation datasets. Furthermore, we reported that the stage-specific prediction models had better predictive power and efficiency after comparing the accuracy, discrimination, calibration and clinical usefulness of stage-specific prediction models and unified models.

## Methods

### Study design and data sources

**Figure 1:**
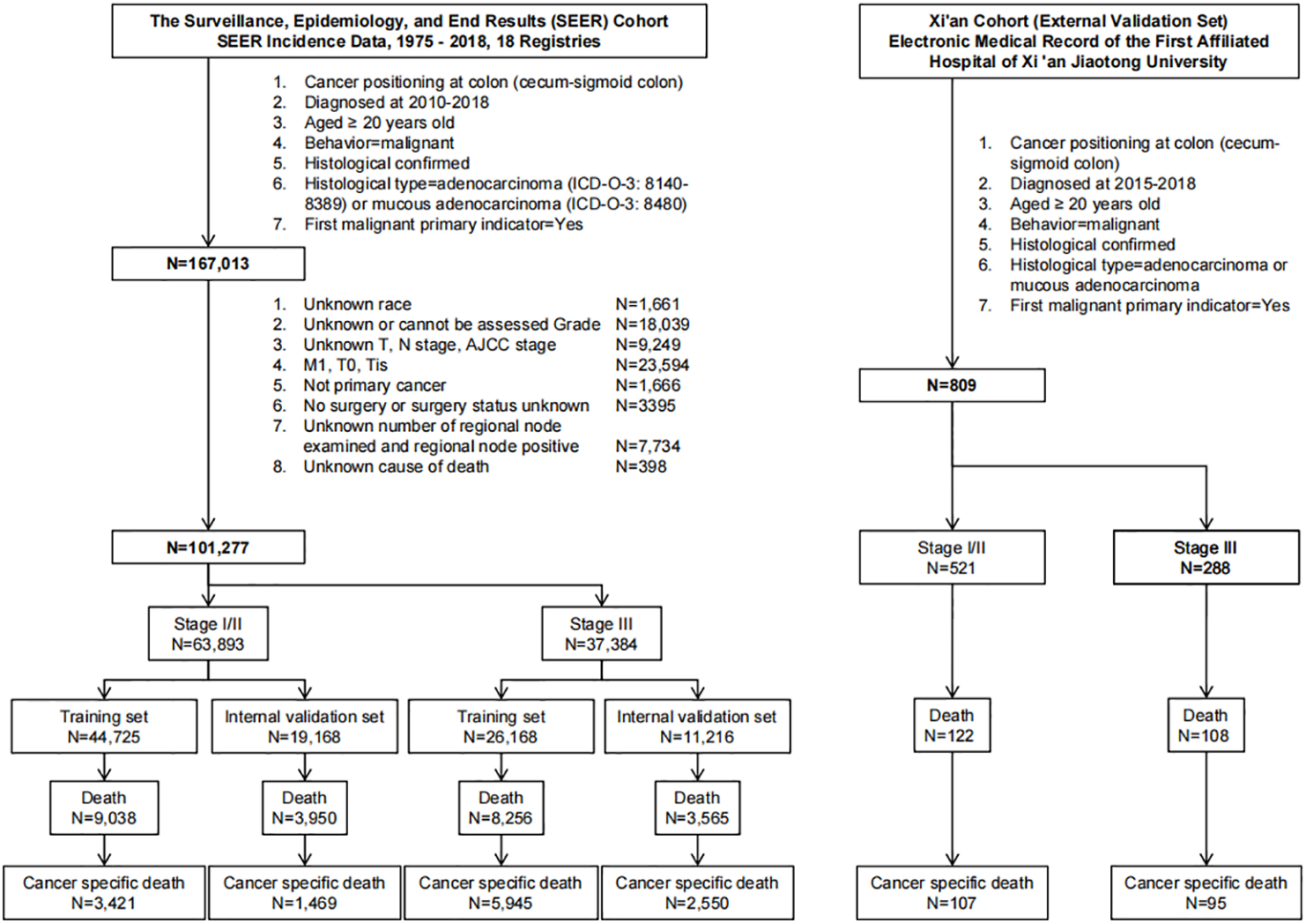
Flowchart for data retrieval and filtration of patients with colon cancer from the SEER database and EMRAS.

We conducted a population-based retrospective cohort study. We established two follow-up cohorts. The main cohort was extracted from the SEER incidence database, which covers approximately 47.9% of the U.S. population. All patients (older than 20 years of age) who were diagnosed with primary colon cancer between 2010 and 2018 and histologically confirmed to have stage I-III malignant adenocarcinoma (ICD-O-3: 8140-8389) or mucous adenocarcinoma (ICD-O-3: 8480) were identified and extracted (**Figure 1**). We excluded patients who met the following criteria: (1) missing demographic information, including age, sex and race; (2) unknown grade and stage, and T0, Tis, M1; (3) no surgery or unknown surgery status; (4) no primary cancer; (5) unknown number of regional nodes examined and regional node positive; and (6) unknown cause of death (**Figure 1**). A total of 101,277 patients were ultimately identified and extracted.

The Xi’an cohort, an external validation cohort, was obtained from the EMRAS database of the First Affiliated Hospital of Xi ‘an Jiaotong University using the same inclusion criteria from 2015 to 2018. A total of 809 patients were ultimately included (**Figure 1**).

We categorized the patients into three groups according to their AJCC stage: Stage I/II group, stage III group and all-stage group. For each group, the training data set was established with 70% of randomly selected patients from the SEER cohort. The remaining 30% of the patients from the SEER cohort were included in the internal validation data set. The Xi’an cohort was defined as an external validation data set (**Figure 1**).

### Outcomes and covariates

For each patient, the baseline was defined as the date of cancer diagnosis. In the Xi’an cohort, patients were followed up until the date of death due to any cause or March 30th, 2020. The outcome of interest was death due to colon cancer in the groups. Death attributed to any other cause was defined as a competing event. We retrieved clinical information, including age, sex, year of diagnosis, race, marital status, tumor position, differentiation grade, histological type, T stage, N stage, radiotherapy, chemotherapy, carcinoembryonic antigen (CEA), tumor deposits, number of examined regional lymph nodes and positive regional lymph nodes and perineural invasion, of each patient from the SEER database or EMRAS database and adjusted for these confounding factors.

### Statistical analyses

All statistical analyses were performed using R statistical software, version 4.0.2 (F Foundation for Statistical Computing, Vienna, Austria). Continuous variables were expressed as the mean ± standard deviation (SD) or median (interquartile range (IQR)) according to the normality of the data. For each group (stage I/II group, stage III group and all-stage group), the least absolute shrinkage and selection operator (LASSO) analysis was used to select the variables. The proportional hazard (PH) assumption for each variable of models were tested before establishing models. *P* value less than 0.05 was considered that such variable violated the PH assumption. For variable violated the PH assumption, we introduced time function (sqrt(t)) to construct interaction term. Considering that death due to other causes was competing for the outcome of interest, we used a competing risk model to estimate and calculate the subdistribution hazard ratio (SHR) and the 95% confidence interval (CI) for cancer-specific death after adjusting for confounding variables selected by the LASSO analysis (10). Nomograms were constructed based on the results of the multivariate competing risk model. The Akaike information criterion (AIC) was used to evaluate the complexity of the model (11). Harrell’s concordance index (C-index) was used to evaluate the accuracy of the prediction (12). The time-dependent receiver operating curve (time-ROC) and time-dependent area under the curve (time-AUC) were used to assess the discrimination of the models. Calibration curves were used to assess the calibration of the models. Decision curve analysis (DCA) was conducted to evaluate the clinical usefulness of the models by calculating the net benefits at different threshold probabilities. X-tile software was used to calculate the cutoff value of the total score of the nomograms (13). All statistical tests were two-sided, and a *P* value less than 0.05 was considered statistically significant.

### Ethical statement

This study was conducted in accordance with the Declaration of Helsinki and was approved by the institutional review board of the First Affiliated Hospital of Xi’an Jiaotong University. All the data from the SEER database were public and deidentified, and individual informed consent was exempted. The data from EMRAS were deidentified, and all patients provided written, broad informed consent at admission. Because this study did not collect new clinical information or biospecimens, additional individual informed consent was exempted.

## Results

### Baseline characteristics of the study cohorts

From the SEER database, we retrieved data for a total of 167,013 patients diagnosed with colon cancer from 2010 to 2018 (**Figure 1**). After exclusion, a total of 101,277 patients were included for further analysis (**Figure 1**). From the EMRAS database, a total of 809 patients diagnosed with colon cancer were retrieved and included (**Figure 1**). In the SEER cohort, the median age at diagnosis was 67 (IQR: 57-77) years. A total of 49.3% (N=49,960) of the patients were male, and 77.8% (N=78,779) of the patients were white. A total of 61.5% (N=62,263) of the patients had tumors located in the right colon. The total follow-up duration was 369,527 person-years, with a median of 3.25 years (IQR: 1.50-5.58 years). In the Xi’an cohort, the median age at diagnosis was 63 (IQR: 54-73) years. A total of 56.6% (N=458) of the patients were male. A total of 52.7% (N=426) of the patients had tumors located in the right colon. The total follow-up duration was 2,219 person-years, with a median of 2.42 years (IQR: 1.33-4.00 years). The detailed demographic, clinicopathological and follow-up information are shown in **Table 1**.

**Table 1:** Baseline characteristics of patients with colon cancer in the training, internal validation and external validation data sets.

Variables	Stage I/II				Stage III			
	SEER cohort			Xi’an cohortN = 521	SEER cohort			Xi’an cohortN = 288
	Training set N = 44,725	Internal validation set N = 19,168	*P*		Training set N = 26,168	Internal validation set N = 11,216	*P*	
Age, years, median (IQR)	68 (59-78)	68 (59-78)	0.838	63 (53-72)	66 (56-76)	65 (55-76)	0.269	64.5 (55-73)
Age, No. (%)			0.388				0.543	
<65	17,377 (38.9)	7,429 (38.8)		296 (56.8)	12,091 (46.2)	5,271 (47.0)		144 (50.0)
≥65	27,348 (61.1)	11,739 (61.2)		225 (43.2)	14,077 (53.8)	5,945 (53.0)		144 (50.0)
Sex, No. (%)			0.835				0.810	
Male	22,129 (49.5)	9,515 (49.6)		315 (60.5)	12,800 (48.9)	5,516 (49.2)		143 (49.7)
Female	22,596 (50.5)	9,653 (50.4)		206 (39.5)	13,368 (51.1)	5,700 (50.8)		145 (50.3)
Year of diagnosis, No. (%)			0.682				0.580	
2010	5,170 (11.6)	2,268 (11.8)		NA	2,930 (11.2)	1,222 (10.9)		NA
2011	5,131 (11.5)	2,244 (11.7)		NA	2,981 (11.4)	1,209 (10.8)		NA
2012	5,168 (11.6)	2,212 (11.5)		NA	2,945 (11.3)	1,258 (11.2)		NA
2013	4,975 (11.1)	2,085 (10.9)		NA	2,956 (11.3)	1,269 (11.3)		NA
2014	5,152 (11.5)	2,214 (11.6)		NA	2,975 (11.4)	1,296 (11.6)		NA
2015	4,977 (11.1)	2,158 (11.3)		133 (25.5)	2,987 (11.4)	1,313 (11.7)		73 (25.3)
2016	5,117 (11.4)	2,143 (11.2)		128 (24.6)	3,015 (11.5)	1,279 (11.4)		71 (24.7)
2017	4,789 (10.7)	2,025 (10.6)		131 (25.1)	2,917 (11.1)	1,304 (11.6)		74 (25.7)
2018	4,246 (9.5)	1,819 (9.5)		129 (24.8)	2,462 (9.4)	1,066 (9.5)		70 (24.3)
Race, No. (%)			0.893				0.747	
White	35,295 (78.9)	15,189 (79.2)		0 (0.0)	19,823 (75.8)	8,472 (75.5)		0 (0.0)
Black	5,324 (11.9)	2,304 (12.0)		0 (0.0)	3,482 (13.3)	1,487 (13.3)		0 (0.0)
Asian or Pacific Islander	3,730 (8.3)	1,545 (8.1)		521 (100.0)	2,662 (10.2)	1,145 (10.2)		288 (100.0)
American Indian/Alaska Native	376 (0.8)	130 (0.7)		0 (0.0)	201 (0.8)	112 (1.0)		0 (0.0)
Marital status, No. (%)			0.461				0.196	
Married (including common law)	23,916 (53.5)	10,069 (52.5)		278 (53.4)	13,804 (52.8)	5,907 (52.7)		152 (52.8)
Others	20,809 (46.5)	9,099 (47.5)		243 (46.6)	12,364 (47.2)	5,309 (47.3)		136 (47.2)
Lateral, No. (%)			0.808				0.304	
Right colon*	28,247 (63.2)	12,114 (63.2)		282 (54.1)	15,359 (58.7)	6,543 (58.3)		144 (50.0)
Left colon*	16,478 (36.8)	7,054 (36.8)		239 (45.9)	10,809 (41.3)	4,673 (41.7)		144 (50.0)
Grade, No. (%)			0.492				0.292	
Well differentiated (Grade I)	5,348 (12.0)	2,282 (11.9)		42 (8.1)	1,413 (5.4)	620 (5.5)		8 (2.8)
Moderately differentiated (Grade II)	33,956 (75.9)	14,551 (75.9)		419 (80.4)	18,322 (70.0)	7,816 (69.7)		216 (75.0)
Poorly differentiated (Grade III)	4,565 (10.2)	1,974 (10.3)		60 (11.5)	5,369 (20.5)	2,318 (20.7)		64 (22.2)
Undifferentiated (Grade IV)	856 (1.9)	361 (1.9)		0 (0.0)	1,064 (4.1)	462 (4.1)		0 (0.0)
Histology, No. (%)			0.182				0.236	
Adenocarcinoma	41,614 (93.0)	17,799 (92.9)		516 (99.0)	24,014 (91.8)	10,299 (91.8)		286 (99.3)
Mucinous adenocarcinoma	3,111 (7.0)	1,369 (7.1)		5 (1.0)	2,154 (8.2)	917 (8.2)		2 (0.7)
T stage, No. (%)			0.079				0.751	
T1	9,685 (21.7)	4,128 (21.5)		32 (6.1)	1,087 (4.2)	473 (4.2)		0 (0.0)
T2	9,150 (20.5)	3,970 (20.7)		41 (7.9)	2,279 (8.7)	922 (8.2)		6 (2.1)
T3	21,887 (48.9)	9,271 (48.4)		86 (16.5)	16,795 (64.2)	7,130 (63.6)		42 (14.6)
T4	4,003 (9.0)	1,799 (9.4)		362 (69.5)	6,007 (23.0)	2,691 (24.0)		240 (83.3)
N stage, No. (%)			NA				0.103	
N0	44,725	19,168		NA	NA	NA		NA
N1	NA	NA		NA	17,740 (67.8)	7,601 (67.8)		185 (64.2)
N2	NA	NA		NA	8,428 (32.2)	3,615 (32.2)		103 (35.8)
Radiotherapy, No. (%) **			0.853				0.755	
None/Unknown	44,339 (99.1)	18,989 (99.1)		462 (88.7)	25,733 (98.3)	11,041 (98.4)		256 (88.9)
Yes	386 (0.9)	179 (0.9)		59 (11.3)	435 (1.7)	175 (1.6)		32 (11.1)
Chemotherapy, No. (%)			0.939				0.227	
None/Unknown	40,266 (90.0)	17,194 (89.7)		382 (73.3)	8,660 (33.1)	3,794 (33.8)		195 (67.7)
Yes	4,459 (10.0)	1,974 (10.3)		139 (26.7)	17,508 (66.9)	7,422 (66.2)		93 (32.3)
CEA, No. (%)			0.532				0.948	
Negative/Borderline	18,007 (40.3)	7,750 (40.4)		227 (43.6)	9,586 (36.6)	4,084 (36.4)		111 (38.5)
Positive	7,871 (17.6)	3,379 (17.6)		123 (23.6)	7,087 (27.1)	3,041 (27.1)		96 (33.3)
Unknown	18,847 (42.1)	8,039 (41.9)		171 (32.8)	9,495 (36.3)	4,091 (36.5)		81 (28.1)
Tumor deposits, No. (%)			0.532				0.831	
No	42,411 (94.8)	18,186 (94.9)		450 (86.4)	19,026 (72.7)	8,113 (72.3)		236 (81.9)
Yes	524 (1.2)	226 (1.2)		71 (13.6)	5,943 (22.7)	2,597 (23.2)		52 (18.1)
Unknown	1,790 (4.0)	756 (3.9)		0 (0.0)	1,199 (4.6)	506 (4.5)		0 (0.0)
Number of examined regional lymph node, median (IQR)	17 (13-24)	17 (13-23.25)	0.707	17 (13-22)	19 (14-25)	19 (14-25)	0.297	16 (12-20)
Number of examined regional lymph node ≥12, No. (%)			0.048				0.560	
<12	5,678 (12.7)	2,417 (12.6)		97 (18.6)	2,378 (9.1)	1,034 (9.2)		56 (19.4)
≥12	39,047 (87.3)	16,751 (87.4)		424 (81.4)	23,790 (90.9)	10,182 (90.8)		232 (80.6)
Number of positive regional lymph node, median (IQR)	NA	NA	NA	NA	2 (1-4)	2 (1-4)	0.231	3 (2-6)
Perineural invasion, No. (%)			0.305				0.833	
No	39,840 (89.1)	17,063 (89.0)		429 (82.3)	20,011 (76.5)	8,552 (76.2)		225 (78.1)
Yes	1,906 (4.3)	824 (4.3)		92 (17.7)	4,358 (16.7)	1,918 (17.1)		63 (21.9)
Unknown	2,979 (6.7)	1,281 (6.7)		0 (0.0)	1,799 (6.9)	746 (6.7)		0 (0.0)
Follow-up time, median (IQR)	43 (20-70)	43 (20-70)	0.605	30 (17-50)	34 (15-62)	34 (15-61)	0.572	25 (15-44.25)
Outcome, No. (%)			0.354^¶^ 0.643^§^				0.359^¶^ 0.643^§^	
Alive	35,687 (79.8)	15,218 (79.4)		399 (76.6)	17,912 (68.5)	7,651 (68.2)		180 (62.5)
Death	9,038 (20.2)	3,950 (20.6)		122 (23.4)	8,256 (31.5)	3,565 (31.8)		108 (37.5)
Cancer-specific death	3,421 (7.6)	1,469 (7.7)		107 (20.5)	5,945 (22.7)	2,550 (22.7)		95 (33.0)
Other cause of deaths	5,617 (12.6)	2,481 (12.9)		15 (2.9)	2,311 (8.8)	1,015 (9.0)		13 (4.5)

*Right colon cancer: cancer at cecum, ascending colon, hepatic flexure, and transverse colon. Left colon cancer: cancer at splenic flexure, descending colon, and sigmoid colon.

**Include beam radiation, radioactive implants, radioisotopes, or combination of these therapy.

^¶^ *P* value of test comparing alive and death between training set and internal validation set.

^§^ *P* value of test comparing alive, cancer-specific death and other cause of death between training set and internal validation set.

NA: data unavailable.

CEA, carcinoembryonic antigen; SEER, The Surveillance, Epidemiology, and End Results.

### Tumor laterality was related to patient prognosis and had different effects on survival in the stage I/II group and stage III group

We first analyzed cancer-specific survival in the stage I and stage II groups (**Table S1**). The results showed similar changes in all the variables between the groups. Because of the small sample size of the stage I group, we combined it with the stage II group into the stage I/II group. 

**Figure 2:**
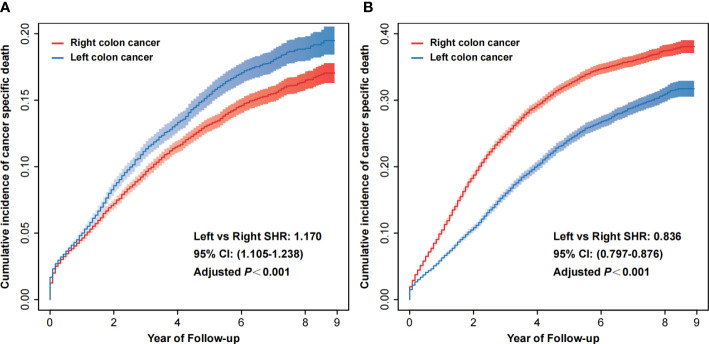
Cumulative incidence of cancer-specific death of right colon cancer and left colon cancer in stage I/II **(A)** and stage III **(B)**. CI, confidence interval; SHR, subdistribution hazard ratio.

According to stage I/II and stage III, we separated the patients retrieved from the SEER database into two groups. We assessed the effects of tumor laterality in these two groups. After excluding the competition for death and adjusting for covariates (age, sex, race, marital status, tumor laterality, differentiation grade, histological type, T stage, N stage, radiotherapy, chemotherapy, CEA, tumor deposits, number of examined regional lymph nodes and perineural invasion), the risk of cancer-specific death was significantly higher in patients with left colon cancer in the stage I/II group (left vs. right SHR: 1.170, 95% CI: 1.105-1.238, adjusted *P*<0.001, **Figure 2A**), while the risk of cancer-specific death was markedly lower in patients with left colon cancer in the stage III group (left vs. right SHR: 0.836, 95% CI: 0.797-0.876, adjusted *P*<0.001, **Figure 2B**). We also observed the same changes in the Xi’an cohort (**Figure S2** and **Table S5**), although there were no statistically significant differences. **Table S6** reported results of PH assumption.

### Variable selection using LASSO analysis

We established three data sets according to the AJCC stage of the patients: The stage I/II, stage III and all-stage groups. For each group, we conducted univariate and multivariate analyses of factors associated with cancer-specific death. In the stage I/II group, age, sex, race, marital status, tumor position, differentiation grade, T-stage, radiotherapy, chemotherapy, CEA, tumor deposits, number of examined regional lymph nodes and perineural invasion were significantly associated with cancer-specific survival (**Table S2**). In the stage III and all-stage groups, all factors were significantly associated with cancer-specific survival (**Table S3** and **Table S4**).

Thus, we conducted LASSO analysis to further reduce the number of variates. According to the results of the LASSO analysis, three lists of variables were established (**Figure S1**). Model 1 included the combination of variables for which the λ value from the LASSO analysis was the minimum value. Model 2 included the most simplified combination of variables for which the λ value from the LASSO analysis was within the minimum value ± 1 standard error (SE). The AJCC model exclusively included T stage and N stage as traditional prognostic prediction models. A total of eight models were established (in the all-stage group, Model 1 and Model 2 included the same lists of variables).

### Establishment of stage-specific prediction models and a unified model and selection of the optimal model

A competing risk model was used to establish the prediction models using the training dataset of each group (**Tables S2**–**S4**). We chose the C-index and AIC to evaluate the accuracy of the three different models to select the optimal model in each group (**Table 2** and **Figure S3**). Furthermore, the time-ROC and time-AUC were used to assess the discriminability of the models (**Figures S4** and **S5**).

**Table 2:** AIC values of different models and C-indexes in predicting cancer-specific survival in the training, internal validation and external validation data sets.

		AIC	C-index		
			Training set	Internal validation set	External validation set
Stage I/II	Model 1*	177960.1	0.731 (0.723-0.739)	0.726 (0.714-0.737)	0.712 (0.641-0.784)
	Model 2**	178045.0	0.730 (0.722-0.739)	0.727 (0.715-0.737)	0.713 (0.640-0.786)
	AJCC model	181356.1	0.637 (0.628-0.646)	0.640 (0.626-0.653)	0.604 (0.531-0.678)
Stage III	Model 1*	152961.7	0.738 (0.728-0.749)	0.741 (0.725-0.758)	0.722 (0.622-0.822)
	Model 2**	152965.0	0.738 (0.727-0.749)	0.741 (0.725-0.757)	0.721 (0.621-0.821)
	AJCC model	156609.7	0.624 (0.612-0.637)	0.612 (0.593-0.631)	0.608 (0.501-0.716)
Stage all***	Model 1*	354346.6	0.702 (0.691-0.713)	0.703 (0.686-0.719)	0.676 (0.574-0.779)
	AJCC model	361503.6	0.589 (0.578-0.601)	0.590 (0.572-0.608)	0.562 (0.485-0.639)

*: Model 1 was established with 15 variables, including age, sex, race, marital status, tumor position, differentiation grade, pathologic type, T stage, N stage (this variable was not included in Model 1 of stage I/II), regional node examined, perineural invasion, tumor deposits, CEA, radiotherapy and chemotherapy, representing the model produced when the λ value of the LASSO regression was minimized.

**: Model 2 was established by selected variables, representing the simplest model when the λ value of the LASSO regression was within lambda.min ± 1 SE. Model 2 for stage I/II was established with 10 variables, including age, sex, race, marital status, tumor position, T stage, regional node examined, perineural invasion, tumor deposits, and CEA. Model 2 for stage III was established with 14 variables, including age, sex, race, marital status, tumor position, differentiation grade, T stage, N stage, regional node examined, perineural invasion, tumor deposits, CEA, radiotherapy and chemotherapy.

***: Model 1 and Model 2 of the all-stage group were identical.

AIC: Akaike information criterion.

**Figure 3:**
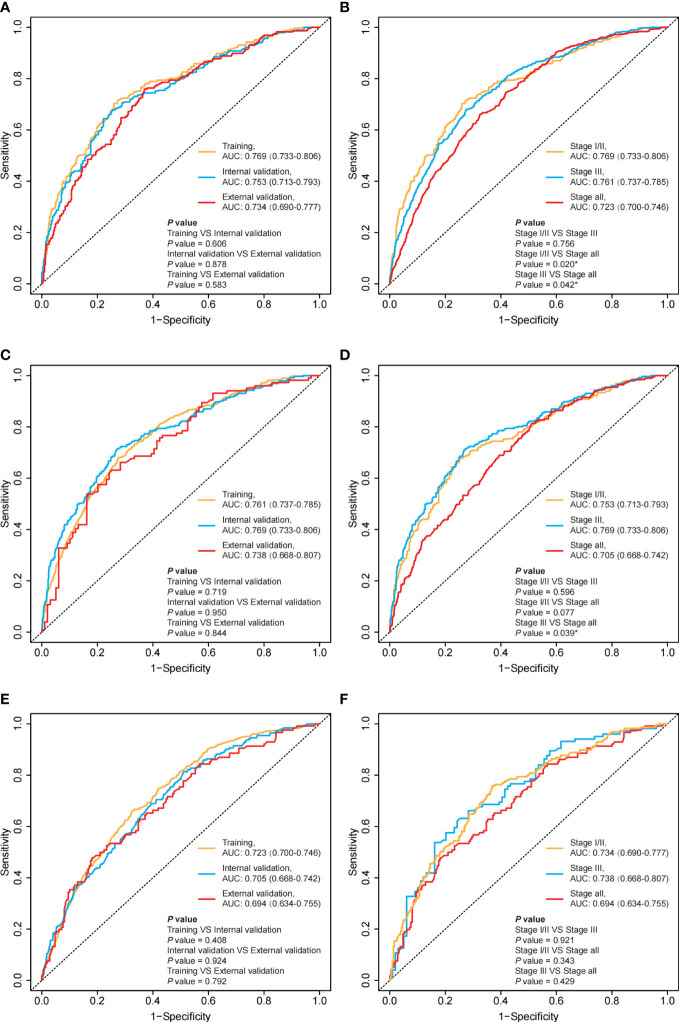
Time-ROC for the training set, internal validation set and external validation set in different groups at the 3^rd^ year and comparing the time-ROC between stage I/II, stage III and all-stage groups. **(A)** the time-ROC for the training set, the internal validation set and the external validation set in the stage I/II group at the 3^rd^ year; **(C)** the time-ROC for the training set, the internal validation set and the external validation set in the stage III group at the 3^rd^ year; **(E)** the time-ROC for the training set, the internal validation set and the external validation set in the all-stage group at the 3^rd^ year; **(B)** the time-ROC comparing the stage-specific prediction models and the unified model in the training set; **(D)** the time-ROC comparing the stage-specific prediction models and the unified model in the internal validation set; **(F)** the time-ROC comparing the stage-specific prediction models and the unified model in the external validation set. *: *P*<0.05.

In the stage I/II group, Model 1 was developed using a combination of 14 variables (**Table S2**), Model 2 was developed using a combination of 10 variables (**Table S2**), and the AJCC model exclusively included T stage. The AIC values and C-indexes were similar between Model 1 and Model 2, but lower in the AJCC model (**Table 2** and **Figure S3**). In addition, the time-ROC and time-AUC showed similar results (**Figures 3**, **S4** and **S5**). However, Model 2 is simpler and easier to use in a clinical setting than Model 1. Therefore, we ultimately chose Model 2 to predict the prognosis of patients with stage I/II colon cancer.

In the stage III group, Model 1 was developed using a combination of 15 variables (**Table S3**), Model 2 was developed using a combination of 14 variables (**Table S3**), and the AJCC model exclusively included T stage and N stage. The AIC values and C-indexes were similar between Model 1 and Model 2 but were lower in the AJCC model (**Table 2** and **Figure S3**). In addition, the time-ROC and time-AUC showed similar results (**Figures 3**, **S4** and **S5**). Therefore, we ultimately chose Model 2 to predict the prognosis of patients with stage III colon cancer.

In the all-stage group, Model 1 was developed using a combination of 15 variables (**Table S4**), and the AJCC model exclusively included T stage and N stage. The AIC values and C-indexes were lower in the AJCC model than in Model 1 (**Table 2** and **Figure S3**). In addition, the time-ROC and time-AUC showed similar results (**Figures 3**, **S4**, **S5**). Therefore, we ultimately chose Model 1 as the unified model to predict the prognosis of patients with colon cancer.

**Figure 4:**
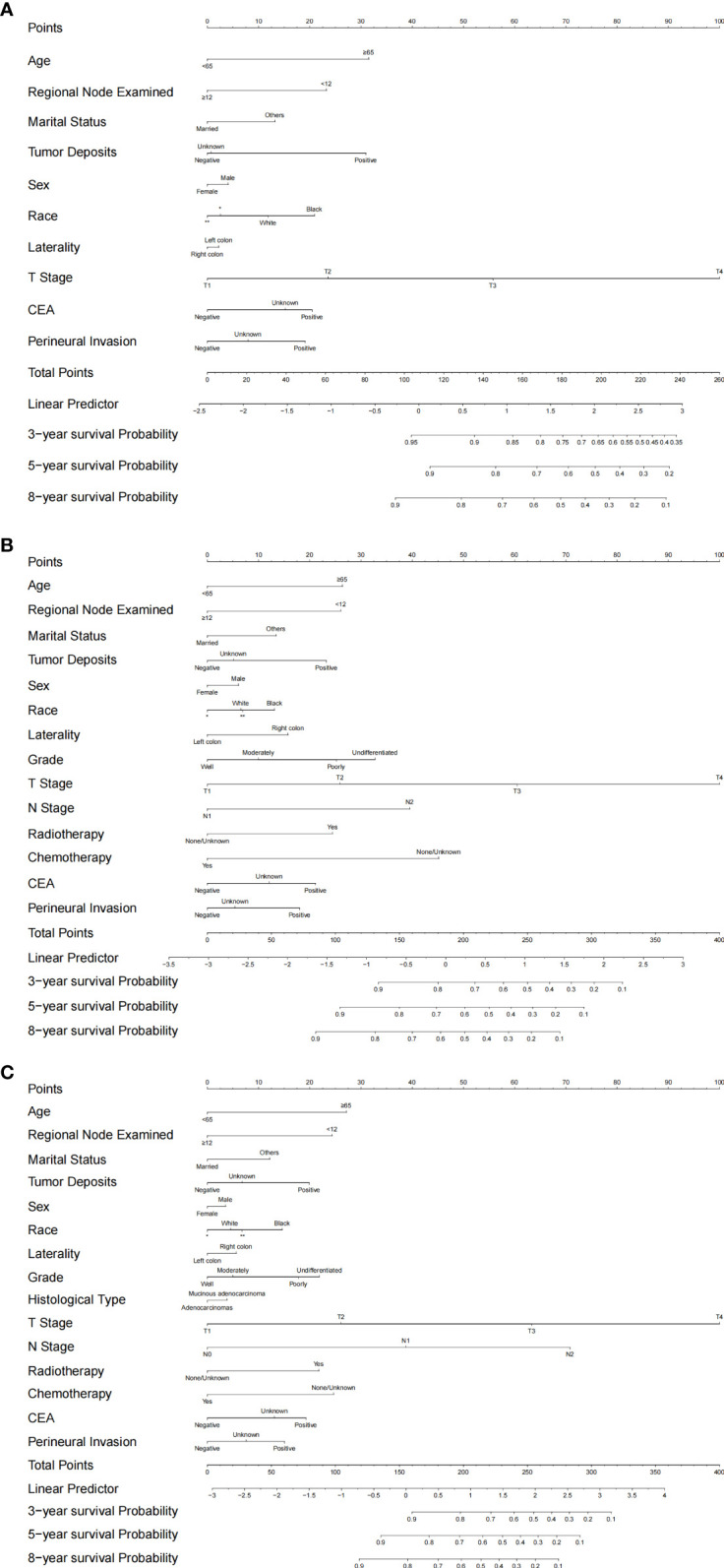
Established nomograms for optimal models for stage I/II, stage III and all-stage groups. **(A)** nomogram for stage I/II; **(B)** nomogram for stage III; **(C)** Nomogram for all stages. *: Asian or Pacific Islander; **: American Indian/Alaska Native.

Based on the selected models, three separate nomograms were established, as shown in **Figure 4**. We estimated the probability of 3-year, 5-year, and 8-year cancer-specific survival.

### The stage-specific prediction models had better performance than the unified model

We evaluated the accuracy of the model predictions using the C-index, as shown in **Table 2**. These three nomograms achieved favorable predictive accuracy. Furthermore, stage-specific prediction models, including the stage I/II prediction model and stage III prediction model, showed better predictive accuracy than the unified model (**Figure S3**).

We assessed the discriminability of the models using the time-ROC and time-AUC (**Figures 3** and **S5**). Except for the external validation set, the 5-year AUC values of the stage-specific prediction models were higher than those of the unified model in the training dataset and the internal validation set (**Figure 3**).

In addition, we assessed the calibration of the models using calibration curves at 3 years (**Figure S6**), 5 years (**Figure S7**) and 8 years (**Figure S8**; the 8-year data were unavailable in the external validation set). Our results showed that the nomograms, including those of the stage-specific prediction models and the unified models, provided optimal agreement between model prediction and actual observations for 3-, 5- and 8-year cancer-specific survival in the training set, internal validation set and external validation set.

Furthermore, we conducted DCA to evaluate the clinical usefulness of the models. Within most of the threshold probability range, the nomograms we established were associated with a higher net benefit. Consistently, the net benefits of the stage-specific models were higher than those of the unified model in predicting 3-year (**Figure S9**), 5-year (**Figure S10**) and 8-year (**Figure S11**; the 8-year data were unavailable in the external validation set) cancer-specific survival in the training set, internal validation set and external validation set.

### Optimal cutoff values of the total score for the stage-specific nomograms

**Figure 5:**
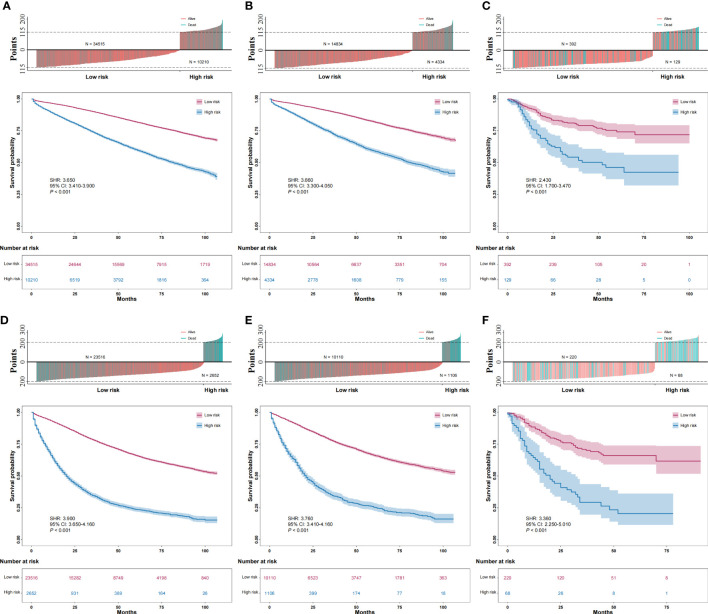
Distribution of total score for patients and survival and comparison of the cancer-specific survival of high-risk and low-risk patients. **(A)**: training data sets of the stage I/II group; **(B)**: internal validation data sets of the stage I/II group; **(C)**: external validation data sets of the stage I/II group; **(D)**: training data sets of the stage III group; **(E)**: internal validation data sets of the stage III group; **(F)**: external validation data sets of the stage III group.

We calculated the optimal cutoff values of the total score for the stage I/II nomogram and stage III nomogram using X-tile software and the training sets of each group. In the stage I/II group, a total score greater than or equal to 115 points was considered high risk. In the stage III group, a total score greater than or equal to 200 points was considered high risk. The distributions of the total scores for patients and cancer-specific survival are shown in **Figure 5**. Furthermore, both in the stage I/II group and stage III group, high-risk patients had worse cancer-specific survival than low-risk patients in the training, internal validation and external validation sets (**Figure 5**).

## Discussion

In this retrospective study, we established two independent cohorts. A total of 101,277 colon cancer patients from the SEER database and 809 colon cancer patients from the EMRAS database were included in the analysis. We confirmed that the laterality of the primary tumor markedly affects the patients’ prognoses, while the effects are contradictory in different stages. We reported that, compared with right-sided colon cancers, the risk of cancer-specific death was higher in patients with left colon cancer in the stage I/II group (left vs. right SHR: 1.170), while the risk of cancer-specific death was markedly lower in patients with left colon cancer in the stage III group (left vs. right SHR: 0.836). Based on the optimal models selected using the LASSO analysis for the groups, we established stage-specific prediction models for stage I/II and stage III separately and a unified prediction model for all stages. The C-index values for the established models were more than 0.7, indicating that the proposed models could correctly predict survival with high accuracy. Moreover, we conducted discrimination and calibration analyses, which indicated that the proposed models were efficient predictors. The results of a DCA indicated that the proposed models could gain higher net benefit within most of the threshold probability range. By validating an independent external validation cohort in a different region and obtaining acceptable results, we reported that the proposed models have high generalizability. However, by comparing the stage-specific prediction models with the unified model, we concluded that the stage-specific prediction models had better predictive power and efficiency. Finally, we calculated the optimal total score cutoff values for the stage-specific nomograms and efficiently identified the high-risk subsets. We can further improve the prediction of survival in colon cancer patients by using stage-specific prediction models.

The laterality of the primary tumor has been widely accepted as one of the independent predictors of tumor prognosis (3). However, whether the prognosis of right-sided colon cancer is better than that of left-sided colon cancer or worse is still controversial and has been challenged by emerging evidence. Recent studies reported an interesting phenomenon in which stage II right-sided colon cancer patients had better survival than left-sided colon cancer patients, and stage III right-sided cancer patients had worse survival (3, 6, 8, 14–17). Weiss et al. reported conflicting results regarding the laterality of the primary tumor for predicting survival at different stages from the SEER database (6). Additionally, researchers also conducted studies and concluded consistent results based on several databases, including the National Cancer Database (NCDB, the United States) and British Columbia Cancer Agency Gastrointestinal Cancer Outcomes Unit (BCCA-GICOU, Canada) (15, 17). Moreover, Kishiki et al. reported that right-sided colon cancers had lower recurrence rates in stage I and stage II patients and a higher recurrence rate in stage III patients according to data retrieved from the databases of 23 institutions belonging to the Japanese Study Group for Postoperative Follow-up of Colorectal Cancer (14). However, although several studies have reported this result, we still lack a strictly evaluated and validated survival prediction tool, considering the different roles of tumor laterality in patients in different stages of colon cancers.

Several studies have shown that left-sided and right-sided colon cancers harbor different clinicopathological, biological and molecular characteristics, which may result in the different contributions of the laterality of primary cancer to survival prediction in the different stages of colon cancer (3). Right-sided and left-sided colon cancer have distinct embryologic origins. Right-sided colon cancer comprising the cecum, the ascending colon and the proximal two-thirds of the transverse colon, derives from the midgut, while the left-sided colon, including the distal one-third of the transverse, the splenic flexure, the descending colon, and the sigmoid colon, derives from the hindgut (18). Due to the distinct origins, the blood supplies of the right-sided and left-sided colon are also different. Branches of the superior mesenteric artery and inferior mesenteric artery mainly perfuse the right-sided and left-sided colon, respectively. Furthermore, such distinct embryologic origin may account for a series of biological and molecular differences between left-sided and right-sided colon cancers. Microsatellite instability (MSI), which is supposed to result from a deficient mismatch repair (MMR) system by either gene mutation or hypermethylation, occurs in approximately 15% of colon cancers and promotes tumorigenesis by generating mutations in target genes that possess coding microsatellite repeats (19, 20). In right-sided colon cancer, MSI was more frequently observed than in left-sided colon cancer (21–23). MSI status is closely related to the survival of patients with colon cancer. Studies have shown that patients with stage II/III MSI colon cancer have better survival than those with microsatellite stability (MSS) (19, 24, 25). However, in contrast, in patients with metastatic colon cancer, the presence of MSI may significantly decrease survival (26). Additionally, the frequency of mutations in key oncogenes and tumor suppressors is significantly different between right-sided and left-sided colon cancers (22). Several key mutations associated with different tumorigenesis pathways and survival, such as BRAF V600E and KRAS, are significantly more common in right-sided colon cancers, while the mutations of APC and TP53 are enriched in left-sided colon cancers (4, 27–31). The differential expression of these key tumor-associated molecules in right-sided versus left-sided colon cancer and their correlation with prognosis may be the result of the distinct embryologic origin of right-sided and left-sided colon cancer and partly explain the difference in prognosis in right-sided versus left-sided colon cancer.

Additionally, right-sided colon cancers, especially hepatic flexure and transverse colon cancers, have the possibility of alternative routes of lymphatic spread through the gastroepiploic ligament (32). Stelzner et al. identified the small blood and lymphatic vessels connecting the transverse colon and the greater omentum and connecting the transverse colon and the pancreas, which may be the potential pathways for lymphatic metastasis to infrapyloric and gastroepiploic lymph nodes (IGLN) (33). Previous studies reported 0.7-22% incidence rates of IGLN metastases for right-sided colon cancer (34–42). In addition, the rates of IGLN metastases were higher (1.7-33%) in patients with positive mesocolic nodes (32). Inadequate dissection of IGLNs could be responsible for two consequences. On the one hand, the residual of possible metastatic nodes may lead to a high rate of local recurrence, however the impact of IGLN metastases on overall survival is still unclear. On the other hand, a lack of assessment of IGLN metastasis results in a misestimation of cancer staging and further affects the choice of adjuvant therapy after surgery. Therefore, several researchers have suggested that extended lymphadenectomy should be performed as a standard treatment for flexure and transverse colon cancers (32). However, if the IGLNs are regional nodes, then IGLN dissection should be performed routinely or selectively, and the exact role of IGLN dissection in prognosis is still unclear.

Risk prediction models have been well used to inform doctors and patients about the risk of disease, the identification of high-risk populations, survival prediction and guiding therapeutic strategies (43, 44). However, an important question is that a model that has a good performance on the training dataset may not perform well when it is applied to another dataset. Model overfitting occurs when too many variables are included (45). Additionally, an established model that uses too many variables is difficult to use. The methods we generally used in the past to select variables were univariable screening and stepwise selection. However, after univariable screening or stepwise selection, the model still includes too many variables and is susceptible to overfitting, especially when the sample size is large. A useful and simple way to reduce overfitting is penalized regression. Two popular penalized regression methods are ridge and LASSO (45). Compared with ridge, LASSO can be used to perform variable selection by shrinking the coefficients to exactly zero. Ambler et al. reported that LASSO is superior to backward elimination and univariable screening when performing variable selection (46). Our results indicated that the 10-variable model performed equally as well as the all-variable models in the stage I/II group, while the univariable analysis showed that all the variables should be included in the model. We effectively reduced the complexity of the developed models by performing variable selection using LASSO.

To our knowledge, this is the first study to develop stage-specific prediction models considering the different effects of the laterality of the primary tumor on colon cancer prognosis at different stages. Our study has several unique strengths. First, a major strength of our study is the use of a large-scale nationwide cohort from the SEER program, a high-quality and reliable database. It allows us to adequately adjust for confounding factors for survival, such as demographic, clinico-pathologic and therapeutic information. Second, we established an external validation dataset from a different race, region and economic and social environment population to validate and evaluate our developed stage-specific prediction models. Finally, to eliminate the potential competitive risks of death due to other reasons, we used a competing risk regression model to calculate SHRs and adjusted for confounding factors.

Several limitations exist in this study and could be a source of bias. First, several pathological information, such as microsatellite status, KRAS mutation and BRAF mutation, are unavailable in the SEER database. While laterality is prognostic, it might be a result of and a surrogate for differences in the molecular factors and/or cancer subtypes between the right-sided and left-sided colon. Although several studies indicated that laterality is independent of molecular factors and cancer subtypes are prognostic factors, the relationship between laterality and molecular markers should be evaluated when developing prediction models (47). Recently, the SEER program has required registries to report the status of some important molecular markers, such as MSI, KRAS and BRAF, as much as possible and may provide these data in the future. Second, the disease-free survival (DFS) data were not captured in the SEER database. Recurrence and metastasis, as important survival outcomes, cannot be evaluated. Third, the detailed information of adjuvant therapy, such as the regimens, dose and completion of chemotherapy and the dose of radiotherapy, was unclear. Finally, we need external validation cohorts of other regions and races to further validate the stage-specific prediction models we developed in this study.

## Conclusions

This study demonstrated that the laterality of the primary tumor affects prognosis in patients with colon cancers, while the effects are contradictory in patients at different stages. Right-sided colon cancer patients have a better survival than left-sided colon cancer patients in stage I/II, while worse survival is observed in stage III patients. We developed stage-specific prediction models considering the contradictory effects of laterality on the precise prediction of cancer-specific survival. By validating in internal and external validation sets, the stage-specific prediction models showed better prediction ability than the unified models and may guide treatment decisions for colon cancer patients.

## Data availability statement

The raw data supporting the conclusions of this article will be made available by the authors, without undue reservation.

## Ethics statement

The studies involving human participants were reviewed and approved by the institutional review board of the First Affiliated Hospital of Xi’an Jiaotong University. Written informed consent for participation was not required for this study in accordance with the national legislation and the institutional requirements.

## Author contributions

CH and FS were responsible for the design of the study, statistical analysis, and drafting and revising of the manuscript; ZZ, LZ, RL, and XS provided critical comments and review of the manuscript; JS and LSZ designed and supervised the study and revised the manuscript. All authors contributed to the article and approved the submitted version.

## References

[1] Sung H Ferlay J Siegel RL Laversanne M Soerjomataram I Jemal A. Global cancer statistics 2020: GLOBOCAN estimates of incidence and mortality worldwide for 36 cancers in 185 countries. Ca-a Cancer J Clin (2021) 71:209–49. doi: 10.3322/caac.21660 33538338

[2] Petrelli F Tomasello G Borgonovo K Ghidini M Turati L Dallera P. Prognostic survival associated with left-sided vs right-sided colon cancer a systematic review and meta-analysis. JAMA Oncol (2017) 3:211–9. doi: 10.1001/jamaoncol.2016.4227 27787550

[3] Lee MS Menter DG Kopetz S. Right versus left colon cancer biology: Integrating the consensus molecular subtypes. J Natl Compr Cancer Netw (2017) 15:411–9. doi: 10.6004/jnccn.2017.0038 28275039

[4] Margonis GA Amini N Buettner S Kim Y Wang J Andreatos N. The prognostic impact of primary tumor site differs according to the KRAS mutational status a study by the international genetic consortium for colorectal liver metastasis. Ann Surg (2021) 273:1165–72. doi: 10.1097/SLA.0000000000003504 31389831

[5] Tejpar S Stintzing S Ciardiello F Tabernero J Van Cutsem E Beier F. Prognostic and predictive relevance of primary tumor location in patients with RAS wild-type metastatic colorectal cancer retrospective analyses of the CRYSTAL and FIRE-3 trials. JAMA Oncol (2017) 3:194–201. doi: 10.1001/jamaoncol.2016.3797 27722750 PMC7505121

[6] Weiss JM Pfau PR O'connor ES King J Loconte N Kennedy G. Mortality by stage for right- versus left-sided colon cancer: Analysis of surveillance, epidemiology, and end results-Medicare data. J Clin Oncol (2011) 29:4401–9. doi: 10.1200/JCO.2011.36.4414 PMC3221523 21969498

[7] Ulanja MB Rishi M Beutler BD Sharma M Patterson DR Gullapalli N. Colon cancer sidedness, presentation, and survival at different stages. J Oncol (2019) 2019:4315032. doi: 10.1155/2019/4315032 30915121 PMC6409047

[8] Warschkow R Sulz MC Marti L Tarantino I Schmied BM Cerny T. Better survival in right-sided versus left-sided stage I - III colon cancer patients. BMC Cancer (2016) 16:554. doi: 10.1186/s12885-016-2412-0 27464835 PMC4964057

[9] Li YQ Feng Y Dai WX Li QG Ca SJ Peng JJ. Prognostic effect of tumor sidedness in colorectal cancer: A SEER-based analysis. Clin Colorectal Cancer (2019) 18:E104–16. doi: 10.1016/j.clcc.2018.10.005 30448100

[10] Fine JP Gray RJ. A proportional hazards model for the subdistribution of a competing risk. J Am Stat Assoc (1999) 94:496–509. doi: 10.1080/01621459.1999.10474144

[11] Akaike H. New look at statistical-model identification. IEEE Trans Automatic Control (1974) 19:716–23. doi: 10.1109/TAC.1974.1100705

[12] Harrell FE Lee KL Mark DB. Multivariable prognostic models: Issues in developing models, evaluating assumptions and adequacy, and measuring and reducing errors. Stat Med (1996) 15:361–87. doi: 10.1002/(SICI)1097-0258(19960229)15:4<361::AID-SIM168>3.0.CO;2-4 8668867

[13] Camp RL Dolled-Filhart M Rimm DL. X-Tile: A new bio-informatics tool for biomarker assessment and outcome-based cut-point optimization. Clin Cancer Res (2004) 10:7252–9. doi: 10.1158/1078-0432.CCR-04-0713 15534099

[14] Kishiki T Kuchta K Matsuoka H Kojima K Asou N Beniya A. The impact of tumor location on the biological and oncological differences of colon cancer: Multi-institutional propensity score-matched study. Am J Surg (2019) 217:46–52. doi: 10.1016/j.amjsurg.2018.07.005 30384969

[15] Kennecke HF Yin Y Davies JM Speers CH Cheung WY Lee-Ying R. Prognostic effect of sidedness in early stage versus advanced colon cancer. Health Sci Rep (2018) 1:e54. doi: 10.1002/hsr2.54 30623090 PMC6266477

[16] Huang ZS Wu JW Li Y Lin YH Li XY. Effect of sidedness on survival among patients with early-stage colon cancer: A SEER-based propensity score matching analysis. World J Surg Oncol (2021) 19:127. doi: 10.1186/s12957-021-02240-3 33874958 PMC8056525

[17] Turner MC Becerra D Sun Z Watson J Leung K Migaly J. The side of the primary tumor affects overall survival in colon adenocarcinoma: An analysis of the national cancer database. Techniques Coloproctology (2019) 23:537–44. doi: 10.1007/s10151-019-01997-w 31190234

[18] Gervaz P Bucher P Morel P. Two colons-two cancers: Paradigm shift and clinical implications. J Surg Oncol (2004) 88:261–6. doi: 10.1002/jso.20156 15565587

[19] Zaanan A Shi Q Taieb J Alberts SR Meyers JP Smyrk TC. Role of deficient DNA mismatch repair status in patients with stage III colon cancer treated with FOLFOX adjuvant chemotherapy a pooled analysis from 2 randomized clinical trials. JAMA Oncol (2018) 4:379–83. doi: 10.1001/jamaoncol.2017.2899 PMC5784452 28983557

[20] Jasmine F Haq Z Kamal M Raza M Da Silva G Gorospe K. Interaction between microsatellite instability (MSI) and tumor DNA methylation in the pathogenesis of colorectal carcinoma. Cancers (2021) 13:4956. doi: 10.3390/cancers13194956 34638440 PMC8508563

[21] Song YL Wang LL Ran WW Li GQ Xiao YJ Wang XN. Effect of tumor location on clinicopathological and molecular markers in colorectal cancer in Eastern China patients: An analysis of 2,356 cases. Front Genet (2020) 11. doi: 10.3389/fgene.2020.00096 PMC7052354 32161617

[22] Muzny DM Bainbridge MN Chang K Dinh HH Drummond JA Fowler G. Comprehensive molecular characterization of human colon and rectal cancer. Nature (2012) 487:330–7. doi: 10.1038/nature11252 PMC3401966 22810696

[23] Sinicrope FA Rego RL Foster N Sargent DJ Windschitl HE Burgart LJ. Microsatellite instability accounts for tumor site-related differences in clinicopathologic variables and prognosis in human colon cancers. Am J Gastroenterol (2006) 101:2818–25. doi: 10.1111/j.1572-0241.2006.00845.x 17026563

[24] Benatti P Gafa R Barana D Marino M Scarselli A Pedroni M. Microsatellite instability and colorectal cancer prognosis. Clin Cancer Res (2005) 11:8332–40. doi: 10.1158/1078-0432.CCR-05-1030 16322293

[25] Taieb J Shi Q Pederson L Alberts S Wolmark N Van Cutsem E. Prognosis of microsatellite instability and/or mismatch repair deficiency stage III colon cancer patients after disease recurrence following adjuvant treatment: results of an ACCENT pooled analysis of seven studies. Ann Oncol (2019) 30:1466–71. doi: 10.1093/annonc/mdz208 PMC7360150 31268130

[26] Venderbosch S Nagtegaal ID Maughan TS Smith CG Cheadle JP Fisher D. Mismatch repair status and BRAF mutation status in metastatic colorectal cancer patients: A pooled analysis of the CAIRO, CAIRO2, COIN, and FOCUS studies. Clin Cancer Res (2014) 20:5322–30. doi: 10.1158/1078-0432.CCR-14-0332 PMC4201568 25139339

[27] Tran B Kopetz S Tie J Gibbs P Jiang ZQ Lieu CH. Impact of BRAF mutation and microsatellite instability on the pattern of metastatic spread and prognosis in metastatic colorectal cancer. Cancer (2011) 117:4623–32. doi: 10.1002/cncr.26086 PMC4257471 21456008

[28] Lochhead P Kuchiba A Imamura Y Liao XY Yamauchi M Nishihara R. Microsatellite instability and BRAF mutation testing in colorectal cancer prognostication. Jnci-Journal Natl Cancer Institute (2013) 105:1151–6. doi: 10.1093/jnci/djt173 PMC3735463 23878352

[29] Tol J Nagtegaal ID Punt CJA. BRAF mutation in metastatic colorectal cancer. New Engl J Med (2009) 361:98–9. doi: 10.1056/NEJMc0904160 19571295

[30] Gonsalves WI Mahoney MR Sargent DJ Nelson GD Alberts SR Sinicrope FA. Patient and tumor characteristics and BRAF and KRAS mutations in colon cancer, NCCTG/Alliance N0147. J Natl Cancer Inst (2014) 106:dju106. doi: 10.1093/jnci/dju106 24925349 PMC4110470

[31] Schell MJ Yang ML Teer JK Lo FY Madan A Coppola D. A multigene mutation classification of 468 colorectal cancers reveals a prognostic role for APC. Nat Commun (2016) 7:11743. doi: 10.1038/ncomms11743 27302369 PMC4912618

[32] Piozzi GN Rusli SM Baek SJ Kwak JM Kim J Kim SH. Infrapyloric and gastroepiploic node dissection for hepatic flexure and transverse colon cancer: A systematic review. Ejso (2022) 48:718–26. doi: 10.1016/j.ejso.2021.12.005 34893366

[33] Stelzner S Hohenberger W Weber K West NP Witzigmann H Wedel T. Anatomy of the transverse colon revisited with respect to complete mesocolic excision and possible pathways of aberrant lymphatic tumor spread. Int J Colorectal Dis (2016) 31:377–84. doi: 10.1007/s00384-015-2434-0 26546443

[34] Toyota S Ohta H Anazawa S. Rationale for extent of lymph-node dissection for right colon-cancer. Dis Colon Rectum (1995) 38:705–11. doi: 10.1007/BF02048026 7607029

[35] Feng B Sun J Ling TL Lu AG Wang ML Chen XY. Laparoscopic complete mesocolic excision (CME) with medial access for right-hemi colon cancer: feasibility and technical strategies. Surg Endoscopy Other Interventional Techniques (2012) 26:3669–75. doi: 10.1007/s00464-012-2435-9 22733200

[36] Feng B Ling TL Lu AG Wang ML Ma JJ Li JW. Completely medial versus hybrid medial approach for laparoscopic complete mesocolic excision in right hemicolon cancer. Surg Endoscopy Other Interventional Techniques (2014) 28:477–83. doi: 10.1007/s00464-013-3225-8 24114515

[37] Bertelsen CA Bols B Ingeholm P Jansen JE Jepsen LV Kristensen B. Lymph node metastases in the gastrocolic ligament in patients with colon cancer. Dis Colon Rectum (2014) 57:839–45. doi: 10.1097/DCR.0000000000000144 24901684

[38] Perrakis A Weber K Merkel S Matzel K Agaimy A Gebbert C. Lymph node metastasis of carcinomas of transverse colon including flexures. consideration of the extramesocolic lymph node stations. Int J Colorectal Dis (2014) 29:1223–9. doi: 10.1007/s00384-014-1971-2 25060216

[39] Uematsu D Akiyama G Sugihara T Magishi A Yamaguchi T Sano T. Laparoscopic radical lymph node dissection for advanced colon cancer close to the hepatic flexure. Asian J Endoscopic Surg (2017) 10:23–7. doi: 10.1111/ases.12311 27515772

[40] Yuksel BC Er S Cetinkaya E Aslar AK. Does transverse colon cancer spread to the extramesocolic lymph node stations?*. Acta Chirurgica Belgica (2021) 121:102–8. doi: 10.1080/00015458.2019.1689642 31701816

[41] Sun YM Zhang DS Feng YF Wang Y Xu ZW Tang JW. Infrapyloric lymph node dissection in right hemicolectomy for colon cancer: Should prophylactic resection be recommended? J Surg Oncol (2021) 123:S30–5. doi: 10.1002/jso.26388 33646617

[42] Wang XJ Huang SH Lu XR Huang Y Chi P. Incidence of and risk factors for gastroepiploic lymph node involvement in patients with cancer of the transverse colon including the hepatic flexure. World J Surg (2021) 45:1514–25. doi: 10.1007/s00268-020-05933-0 33475804

[43] Moons KGM Royston P Vergouwe Y Grobbee DE Altman DG. Prognosis and prognostic research: what, why, and how? Bmj-British Med J (2009) 338:b375. doi: 10.1136/bmj.b375 19237405

[44] Moons KGM Kengne AP Woodward M Royston P Vergouwe Y Altman DG. Risk prediction models: I. development, internal validation, and assessing the incremental value of a new (bio)marker. Heart (2012) 98:683–90. doi: 10.1136/heartjnl-2011-301246 22397945

[45] Pavlou M Ambler G Seaman S De Iorio M Omar RZ. Review and evaluation of penalised regression methods for risk prediction in low-dimensional data with few events. Stat Med (2016) 35:1159–77. doi: 10.1002/sim.6782 PMC4982098 26514699

[46] Ambler G Seaman S Omar RZ. An evaluation of penalised survival methods for developing prognostic models with rare events. Stat Med (2012) 31:1150–61. doi: 10.1002/sim.4371 21997569

[47] Venook AP Ou FS Lenz HJ Kabbarah O Qu XP Niedzwiecki D. Primary (1 degrees) tumor location as an independent prognostic marker from molecular features for overall survival (OS) in patients (pts) with metastatic colorectal cancer (mCRC): Analysis of CALGB / SWOG 80405 (Alliance). J Clin Oncol (2017) 35:3503. doi: 10.1200/JCO.2017.35.15_suppl.3503

