# Supplementary material for: Development and validation of a new stage-specific nomogram model for predicting cancer-specific survival in patients in different stages of colon cancer: A SEER population-based study and external validation
Source: Front Oncol. 2022 Dec 7;12:1024467. doi: 10.3389/fonc.2022.1024467 (PMC9768485; doi:10.3389/fonc.2022.1024467)
Supplement: Supplementary file 1 [file DataSheet_1.docx]

***Supplementary Material***


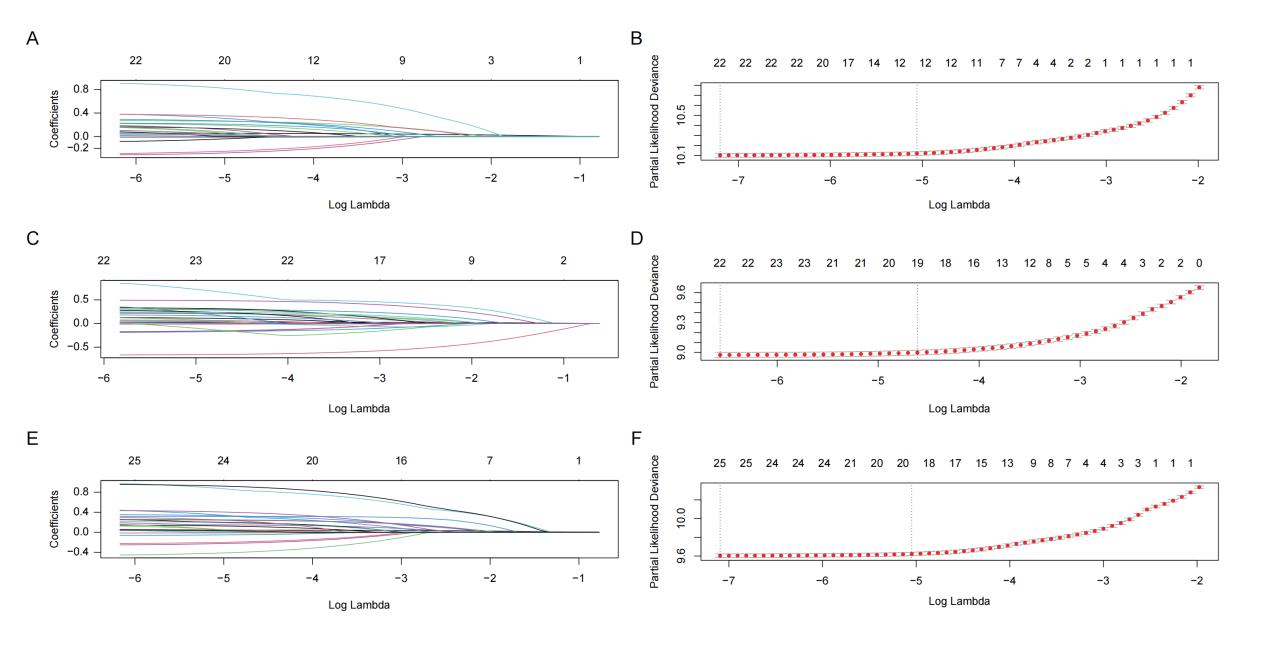


**Figure S1: Variable selection using LASSO analysis.** (A), (C) and (E): coefficient profile plot for stage I/II, stage III and all-stage groups, respectively. (B), (D) and (F): 10-fold cross-validation curve for stage I/II, stage III and all-stage groups, respectively.


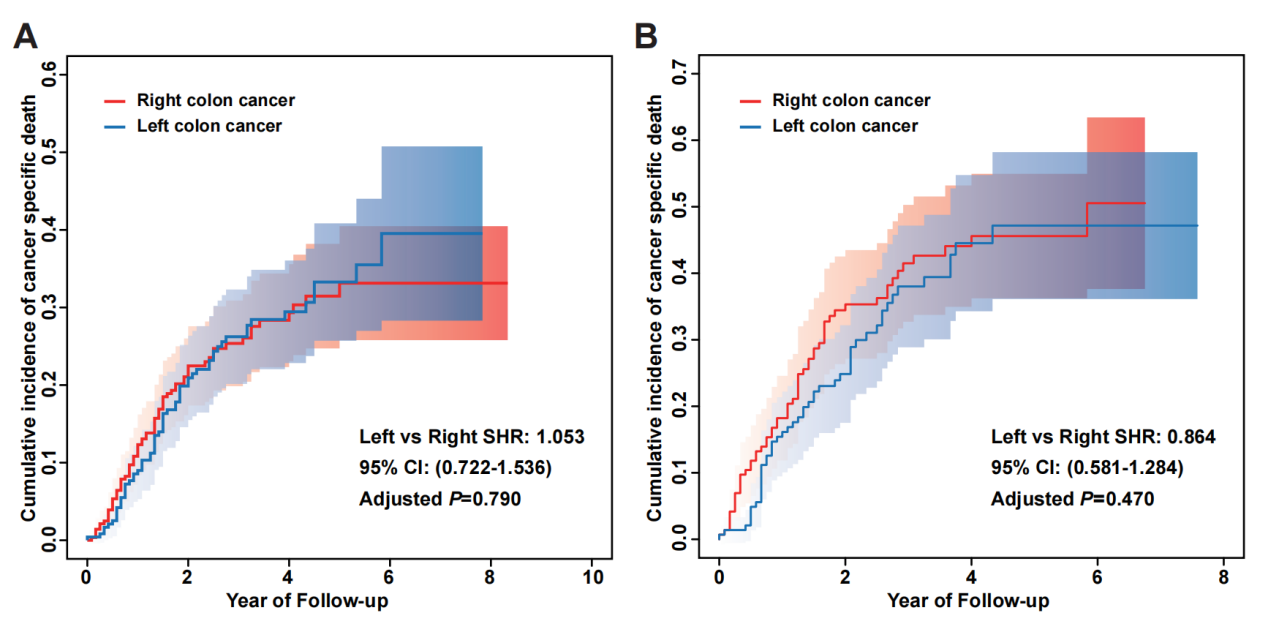


**Figure S2: Cumulative incidence of cancer-specific death of right colon cancer and left colon cancer in stage I/II (A) and stage III (B) of Xi’an cohort.** CI, confidence interval; SHR, subdistribution hazard ratio.


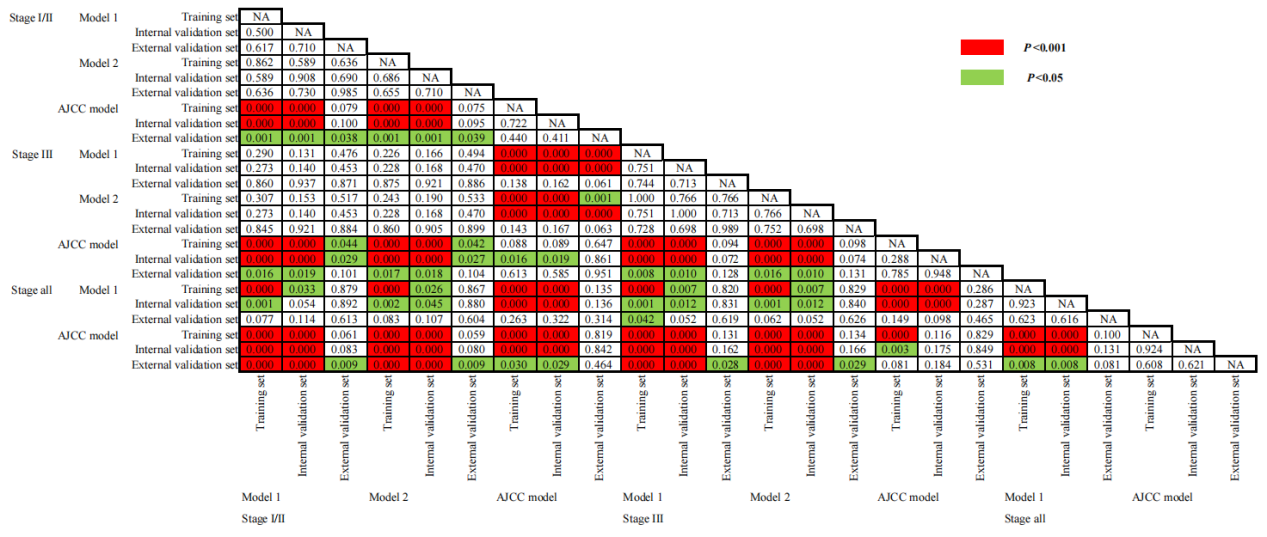


**Figure S3: P values of the C-indexes in predicting cancer-specific survival in the training, internal validation and external validation data sets.**


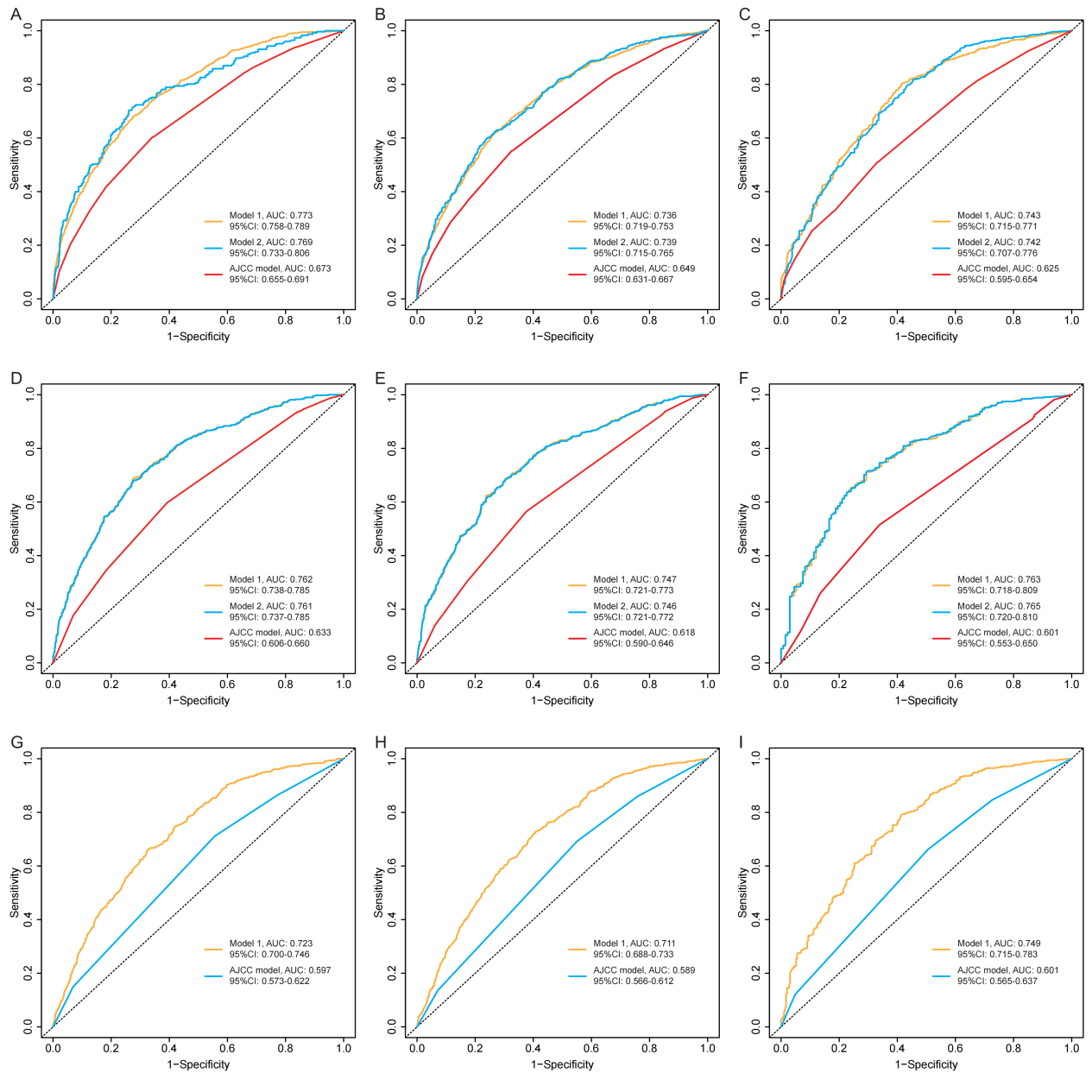


**Figure S4: Time-ROC for different developed models of stage I/II, stage III and all-stage groups.** (A): the time-ROC for the different models in the stage I/II group at the 3^rd^ year; (B): the time-ROC for the different models in the stage I/II group at the 5^th^ year; (C): the time-ROC for the different models in the stage I/II group at the 8^th^ year; (D): the time-ROC for the different models in the stage III group at the 3^rd^ year; (E): the time-ROC for the different models in the stage III group at the 5^th^ year; (F): the time-ROC for the different models in the stage III group at the 8^th^ year; (G): the time-ROC for the different models in the all-stage group at the 3^rd^ year; (H): the time-ROC for the different models in the all-stage group at the 5^th^ year; (I): the time-ROC for the different models in the all-stage group at the 8^th^ year.


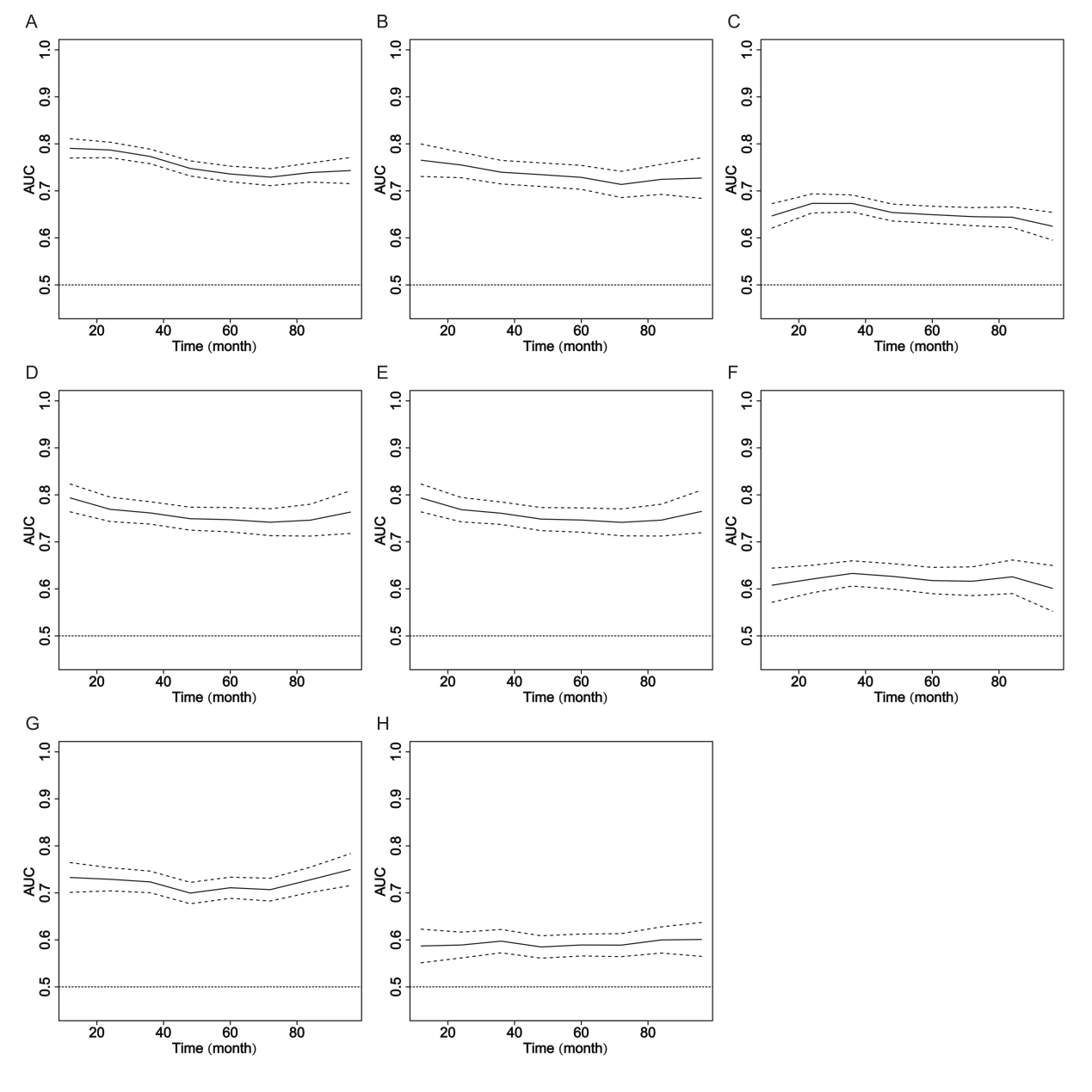


**Figure S5: Time-AUC for different developed models of the stage I/II, the stage III and the all-stage groups.** (A), (B) and (C): the time-AUC for Model 1, Model 2 and AJCC model in the stage I/II group, respectively; (D), (E) and (F): the time-AUC for Model 1, Model 2 and AJCC model in the stage III group, respectively; (G) and (H): the time-AUC for Model 1 and Model 2 in the all-stage group.


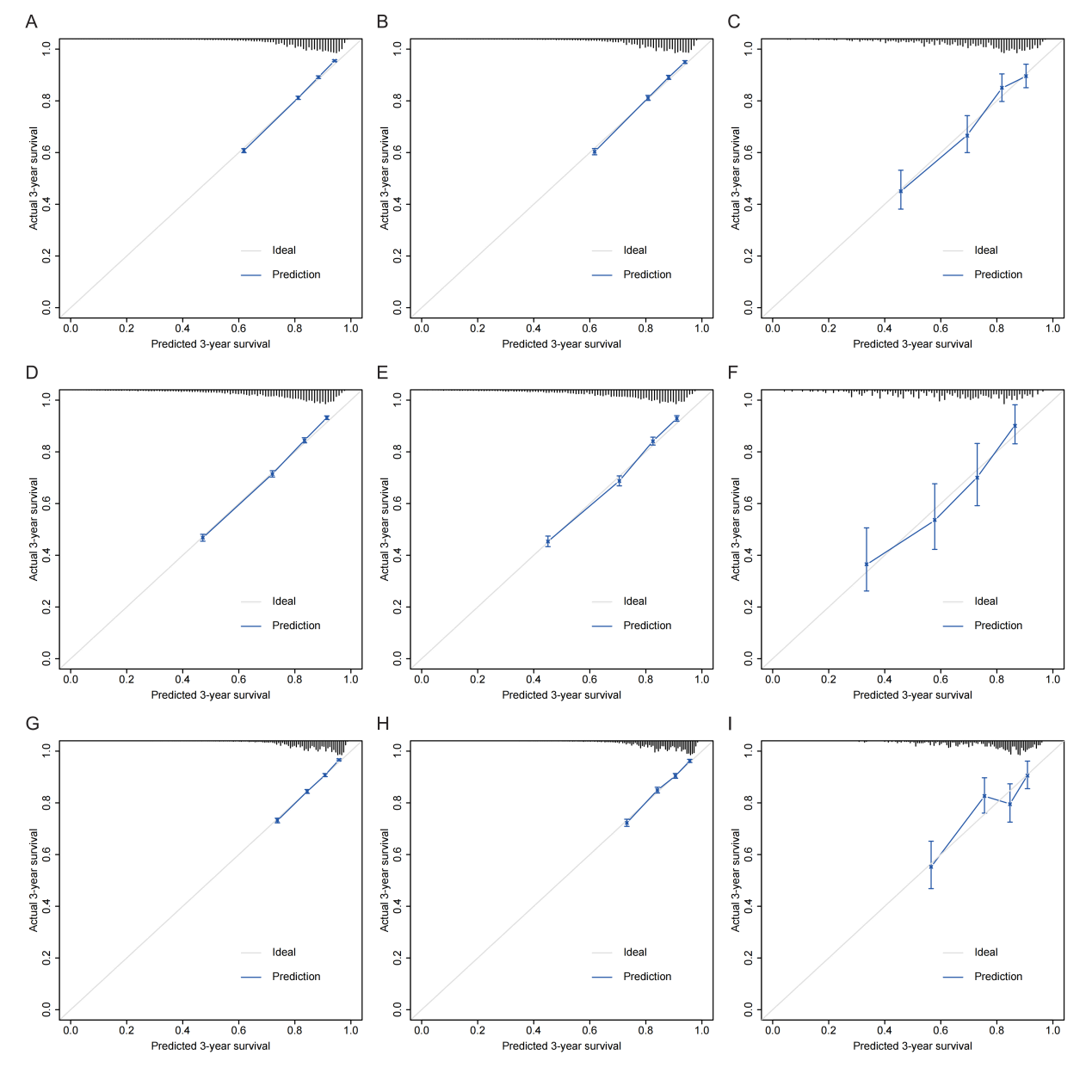


**Figure S6:** **Calibration curves of models in the training set, internal validation set and external validation set of stage I/II, stage III and all-stage groups at the 3^rd^ year.** (A), (B) and (C): The calibration curve of the model in the training set, the internal validation set and the external validation set of the stage I/II group at the 3^rd^ year, respectively. (D), (E) and (F): The calibration curve of the model in the training set, the internal validation set and the external validation set of the stage III group at the 3^rd^ year, respectively. (G), (H) and (I): The calibration curve of the model in the training set, the internal validation set and the external validation set of the all-stage group at the 3^rd^ year, respectively.


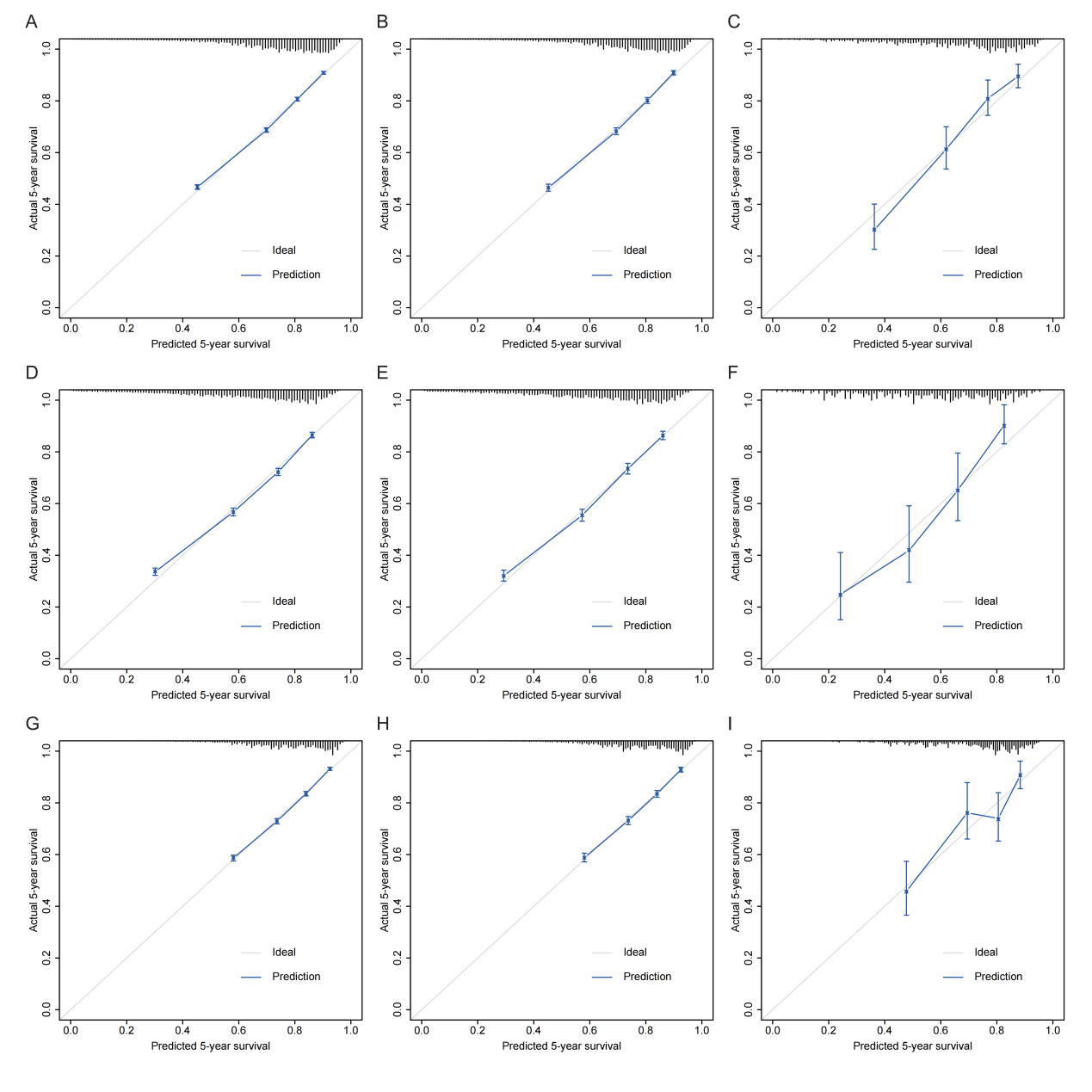


**Figure S7:** **Calibration curves of models in the training set, internal validation set and external validation set of stage I/II, stage III and all-stage groups at the 5^th^ year.** (A), (B) and (C): The calibration curve of the model in the training set, the internal validation set and the external validation set of the stage I/II group at the 5^th^ year, respectively. (D), (E) and (F): The calibration curve of the model in the training set, the internal validation set and the external validation set of the stage III group at the 5^th^ year, respectively. (G), (H) and (I): The calibration curve of the model in the training set, the internal validation set and the external validation set of the all-stage group at the 5^th^ year, respectively.


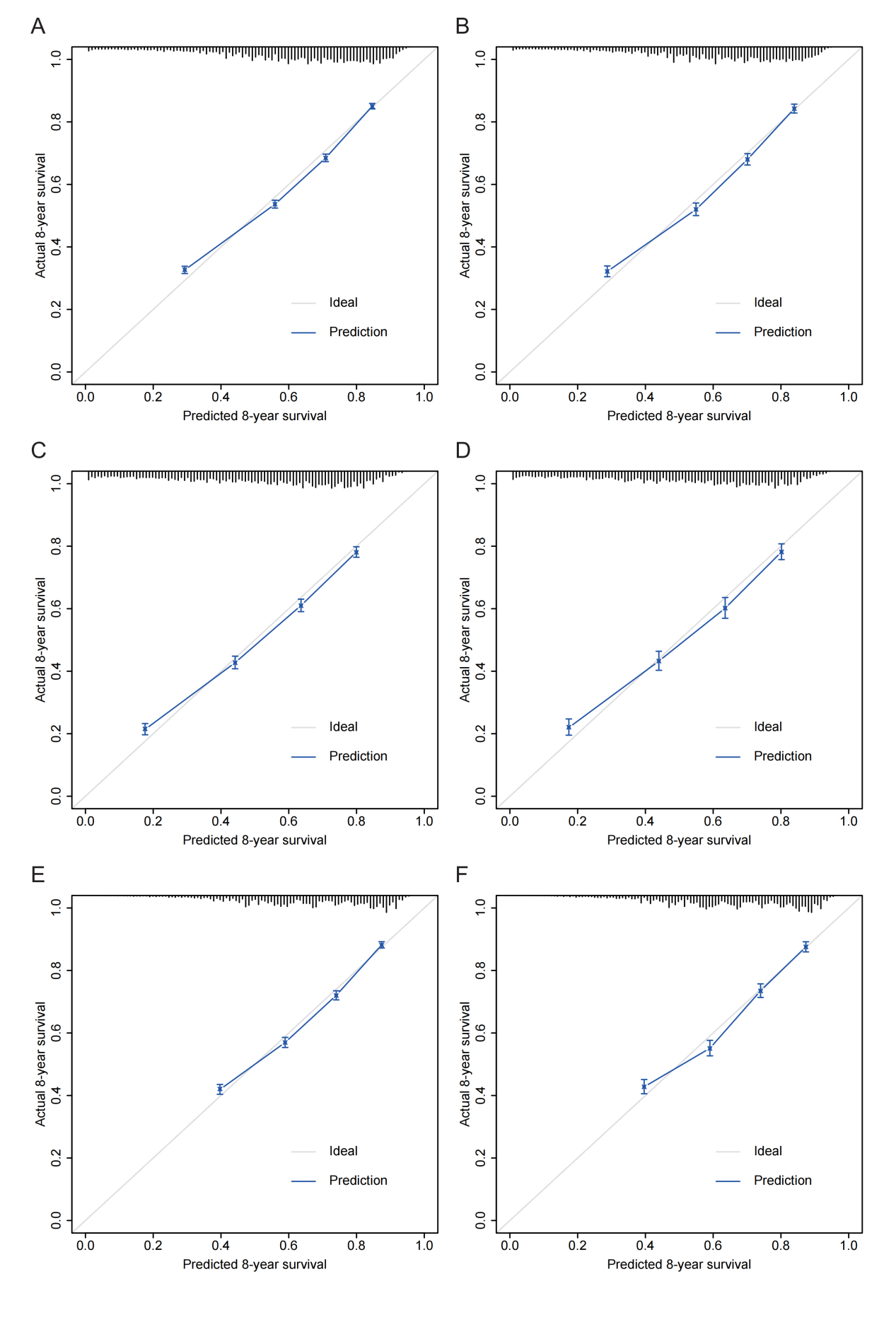


**Figure S8:** **Calibration curves of models in the training set, internal validation set of stage I/II, stage III and all-stage groups at the 8^th^ year.** (A) and (B): The calibration curve of the model in the training set and the internal validation set of the stage I/II group at the 8^th^ year, respectively. (C) and (D): The calibration curve of the model in the training set and the internal validation set of the stage III group at the 8^th^ year, respectively. (E) and (F): The calibration curve of the model in the training set and the internal validation set of the all-stage group at the 8^th^ year, respectively.


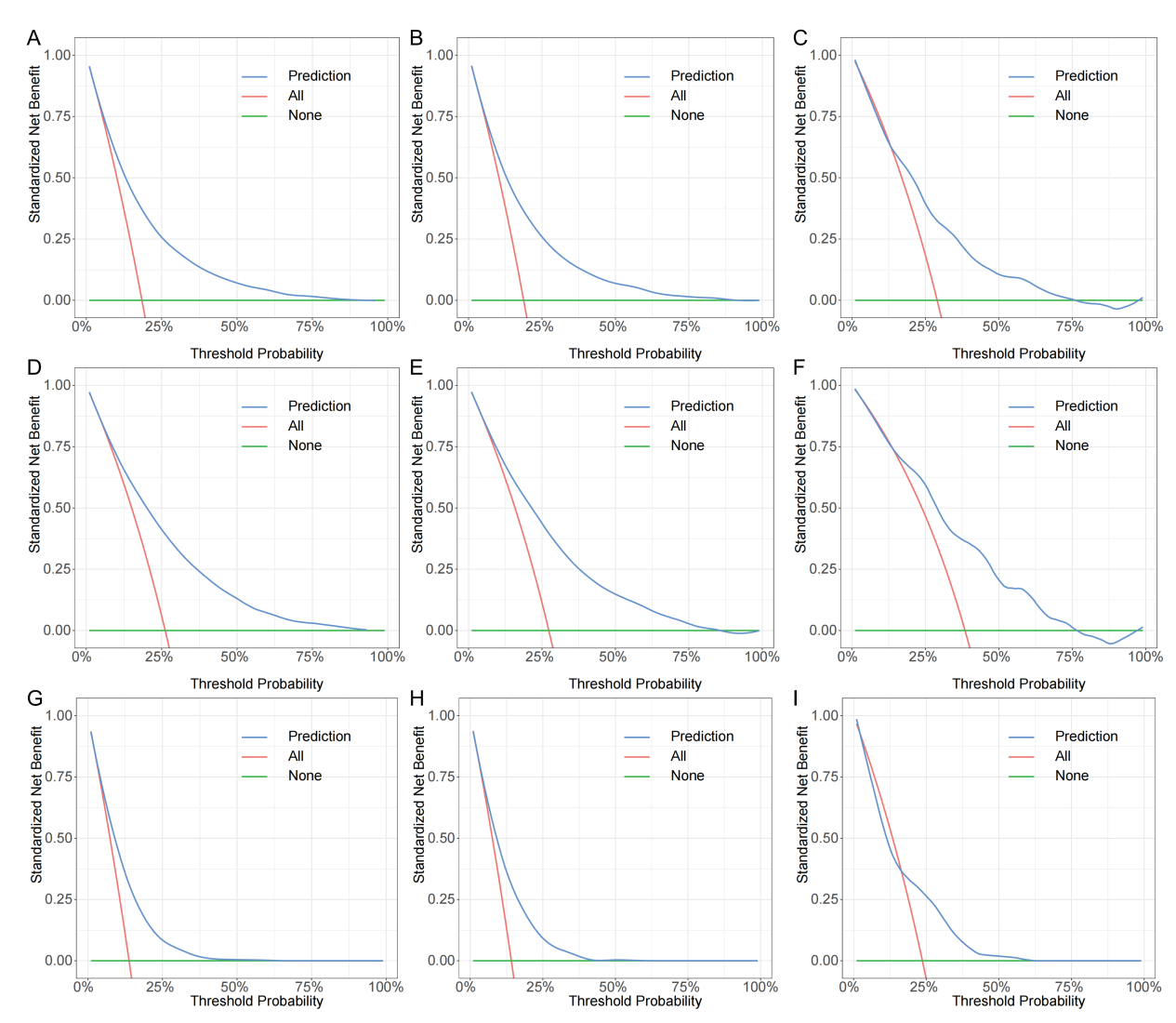


**Figure S9:** **DCA of the models in the training set, the internal validation set and the external validation set of the stage I/II, the stage III and the all-stage groups at the 3^rd^ year.** (A), (B) and (C): DCA of the model in the training set, the internal validation set and the external validation set of the stage I/II group at the 3^rd^ year, respectively. (D), (E) and (F): DCA of the model in the training set, the internal validation set and the external validation set of the stage III group at the 3^rd^ year, respectively. (G), (H) and (I): DCA of the model in the training set, the internal validation set and the external validation set of the all-stage group at the 3^rd^ year, respectively.


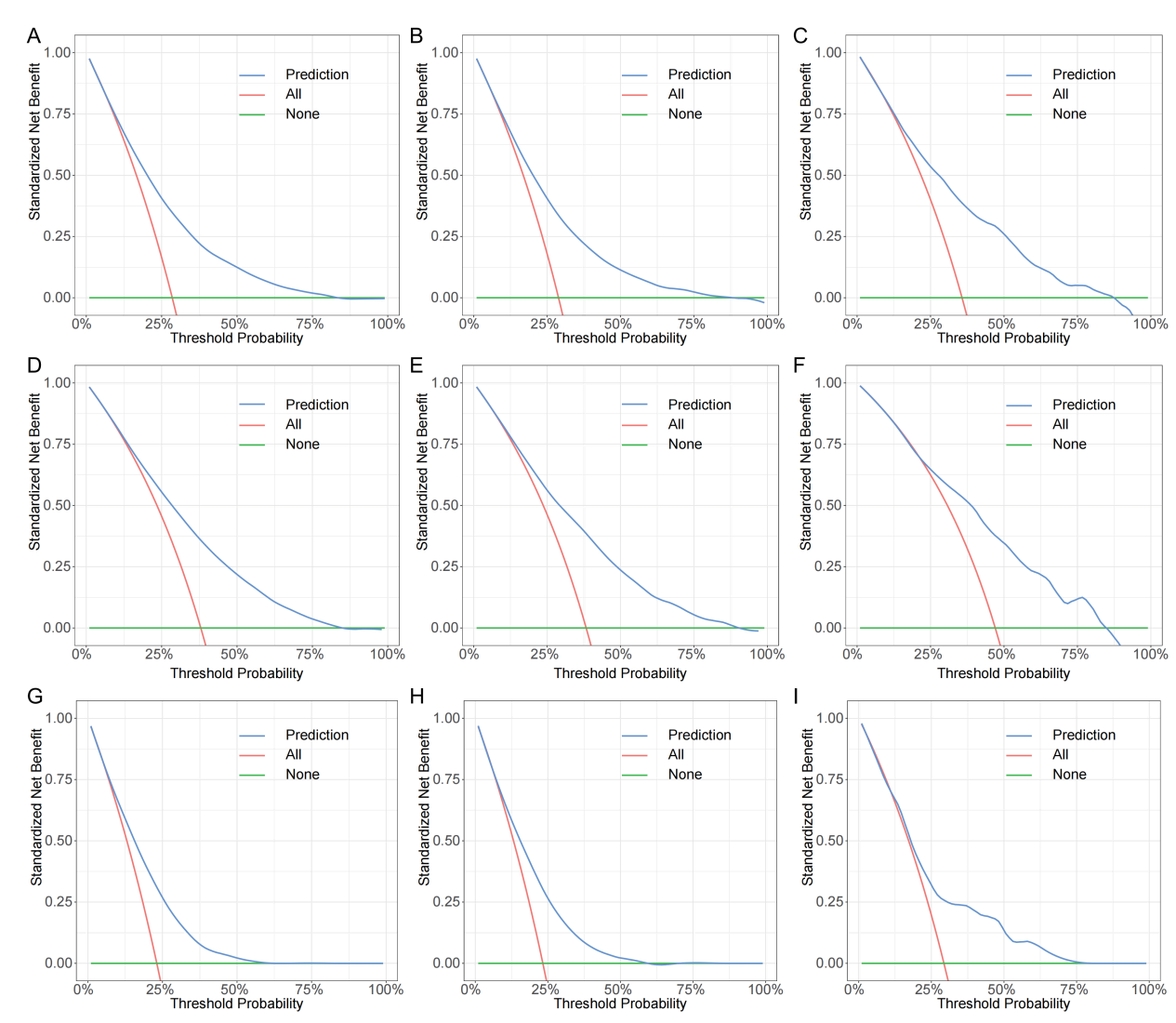


**Figure S10:** **DCA of the models in the training set, the internal validation set and the external validation set of the stage I/II, the stage III and the all-stage groups at the 5^th^ year.** (A), (B) and (C): DCA of the model in the training set, the internal validation set and the external validation set of the stage I/II group at the 5^th^ year, respectively. (D), (E) and (F): DCA of the model in the training set, the internal validation set and the external validation set of the stage III group at the 5^th^ year, respectively. (G), (H) and (I): DCA of the model in the training set, the internal validation set and the external validation set of the all-stage group at the 5^th^ year, respectively.


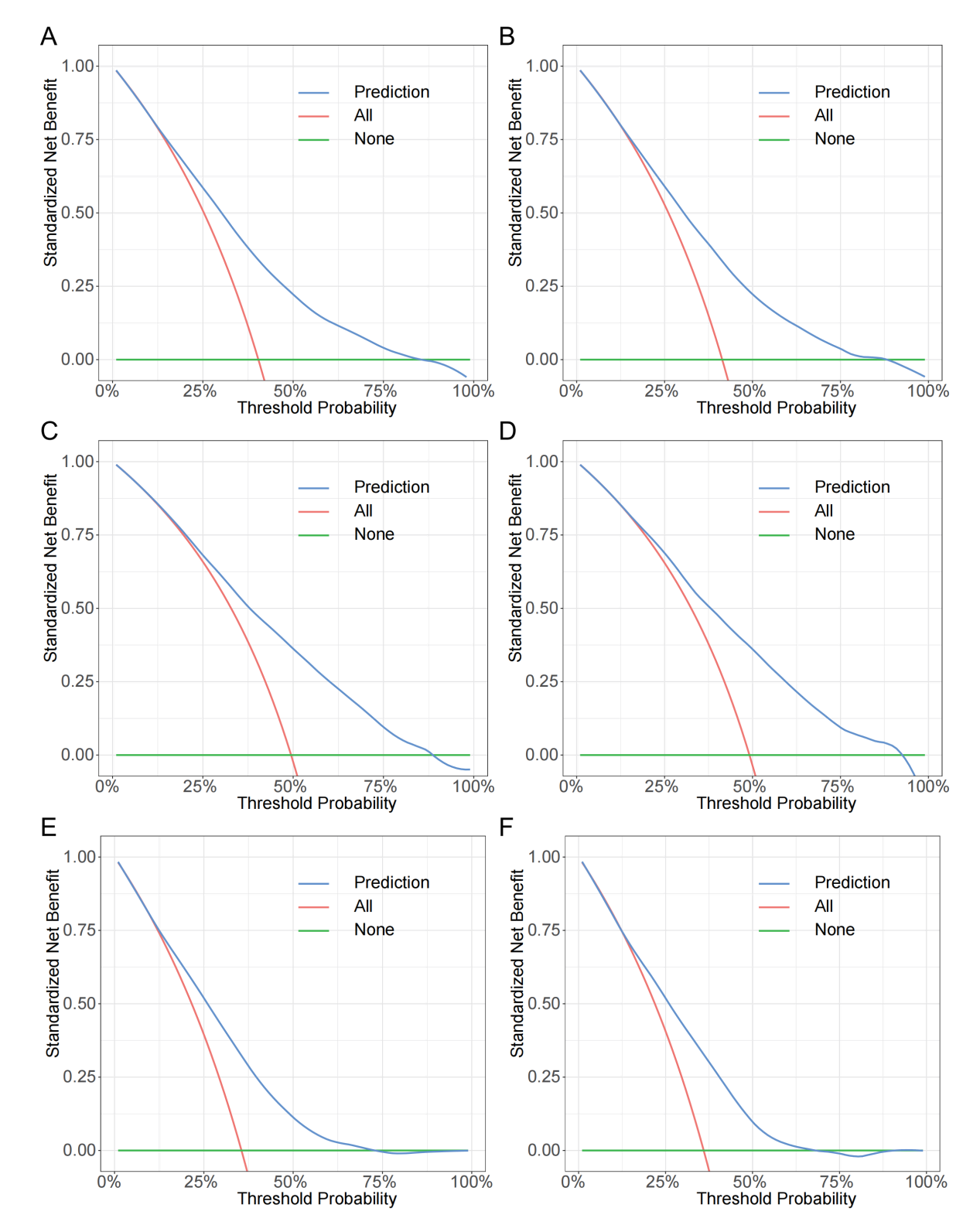


**Figure S11:** **DCA of models in the training set, the internal validation set of the stage I/II, the stage III and the all-stage groups at the 8^th^ year.** (A) and (B): DCA of the model in the training set and the internal validation set of the stage I/II group at the 8^th^ year, respectively. (C) and (D): DCA of the model in the training set and the internal validation set of the stage III group at the 8^th^ year, respectively. (E) and (F): DCA of the model in the training set and the internal validation set of the all-stage group at the 8^th^ year, respectively.

**Table S1. Multivariate analysis of cancer-specific death for patients in stage I and II groups.**

| Variables | | Multivariate analysis | | | |
| --- | --- | --- | --- | --- | --- |
|  |  | Stage I | | Stage II | |
|  |  | SHR (95% CI) | *P* | SHR (95% CI) | *P* |
| Age, No. (%) | |  |  |  |  |
|  | <65 | Ref. |  | Ref. |  |
|  | ≥65 | 2.629 (2.163-3.196) | <0.001 | 1.762 (1.612-1.926) | <0.001 |
| Sex, No. (%) | |  |  |  |  |
|  | Male | Ref. |  | Ref. |  |
|  | Female | 0.859 (0.730-1.011) | 0.067 | 0.942 (0.872-1.017) | 0.130 |
| Race, No. (%) | |  |  |  |  |
|  | White | Ref. |  | Ref. |  |
|  | Black | 1.342 (1.075-1.674) | 0.009 | 1.212 (1.084-1.356) | <0.001 |
|  | Asian or Pacific Islander | 0.778 (0.558-1.085) | 0.140 | 0.830 (0.712-0.968) | 0.018 |
|  | American Indian/Alaska Native | 1.070 (0.400-2.865) | 0.890 | 0.717 (0.433-1.188) | 0.200 |
| Marital status, No. (%) | |  |  |  |  |
|  | Married (including common law) | Ref. |  | Ref. |  |
|  | Others | 1.276 (1.085-1.501) | 0.003 | 1.316 (1.218-1.423) | <0.001 |
| Lateral, No. (%) | |  |  |  |  |
|  | Right colon* | Ref. |  | Ref. |  |
|  | Left colon* | 1.100 (0.944-1.264) | 0.160 | 1.189 (1.104-1.282) | <0.001 |
| Grade, No. (%) | |  |  |  |  |
|  | Well differentiated (Grade I) | Ref. |  | Ref. |  |
|  | Moderately differentiated (Grade II) | 1.104 (0.882-1.383) | 0.390 | 1.066 (0.918-1.239) | 0.400 |
|  | Poorly differentiated (Grade III) | 1.130 (0.790-1.618) | 0.500 | 1.275 (1.071-1.518) | 0.006 |
|  | Undifferentiated (Grade IV) | 1.416 (0.706-2.840) | 0.330 | 1.081 (0.824-1.419) | 0.570 |
| Histology, No. (%) | |  |  |  |  |
|  | Adenocarcinoma | Ref. |  | Ref. |  |
|  | Mucinous adenocarcinoma | 1.200 (0.852-1.691) | 0.300 | 0.939 (0.825-1.068) | 0.340 |
| T stage, No. (%) | |  |  |  |  |
|  | T1 | Ref. |  | - |  |
|  | T2 | 1.493 (1.265-1.762) | <0.001 | - |  |
|  | T3 | - |  | Ref. |  |
|  | T4 | - |  | 2.624 (2.402-2.866) | <0.001 |
| Radiotherapy, No. (%) ** | |  |  |  |  |
|  | None/Unknown | Ref. |  | Ref. |  |
|  | Yes | 0.376 (0.041-3.484) | 0.390 | 1.322 (1.056-1.656) | 0.015 |
| Chemotherapy, No. (%) | |  |  |  |  |
|  | None/Unknown | Ref. |  | Ref. |  |
|  | Yes | 3.028 (1.676-5.472) | <0.001 | 0.911 (0.816-1.017) | 0.096 |
| CEA, No. (%) | |  |  |  |  |
|  | Negative/Borderline | Ref. |  | Ref. |  |
|  | Positive | 1.898 (1.501-2.399) | <0.001 | 1.523 (1.377-1.684) | <0.001 |
|  | Unknown | 1.218 (1.022-1.450) | 0.027 | 1.444 (1.318-1.582) | <0.001 |
| Tumor deposits, No. (%) | |  |  |  |  |
|  | No | Ref. |  | Ref. |  |
|  | Yes | 0.974 (0.314-3.025) | 0.960 | 2.024 (1.684-2.433) | <0.001 |
|  | Unknown | 0.865 (0.573-1.305) | 0.490 | 1.056 (0.874-1.276) | 0.570 |
| Number of examined regional lymph node ≥12, No. (%) | |  |  |  |  |
|  | <12 | Ref. |  | Ref. |  |
|  | ≥12 | 0.779 (0.644-0.942) | 0.010 | 0.546 (0.493-0.606) | <0.001 |
| Perineural invasion, No. (%) | |  |  |  |  |
|  | No | Ref. |  | Ref. |  |
|  | Yes | 0.783 (0.369-1.659) | 0.520 | 1.541 (1.352-1.757) | <0.001 |
|  | Unknown | 1.190 (0.894-1.582) | 0.230 | 1.230 (1.068-1.417) | 0.004 |

*Right colon cancer: cancer at cecum, ascending colon, hepatic flexure, and transverse colon. Left colon cancer: cancer at splenic flexure, descending colon, and sigmoid colon.

**Include beam radiation, radioactive implants, radioisotopes, or combination of these therapy.

CEA: carcinoembryonic antigen.

**Table S2. Univariate and multivariate analyses of cancer-specific death for patients in the stage I/II group.**

| Variables | | Univariate analysis | | Multivariate analysis | | | | | |
| --- | --- | --- | --- | --- | --- | --- | --- | --- | --- |
|  |  |  |  | Model 1 | | Model 2 | | AJCC model | |
|  |  | SHR (95% CI) | *P* | SHR (95% CI) | *P* | SHR (95% CI) | *P* | SHR (95% CI) | *P* |
| Age, No. (%) | |  |  |  |  |  |  |  |  |
|  | <65 | Ref. |  | Ref. |  | Ref. |  |  |  |
|  | ≥65 | 1.970 (1.820-2.130) | <0.001 | 1.915 (1.766-2.076) | <0.001 | 1.925 (1.779-2.083) | <0.001 |  |  |
| Sex, No. (%) | |  |  |  |  |  |  |  |  |
|  | Male | Ref. |  | Ref. |  | Ref. |  |  |  |
|  | Female | 1.050 (0.982-1.120) | 0.150 | 0.884 (0.824-0.949) | 0.001 | 0.888 (0.827-0.953) | 0.001 |  |  |
| Race, No. (%) | |  |  |  |  |  |  |  |  |
|  | White | Ref. |  | Ref. |  | Ref. |  |  |  |
|  | Black | 1.183 (1.073-1.304) | 0.001 | 1.322 (1.195-1.462) | <0.001 | 1.310 (1.184-1.448) | <0.001 |  |  |
|  | Asian or Pacific Islander | 0.795 (0.693-0.913) | 0.001 | 0.842 (0.733-0.968) | 0.015 | 0.837 (0.729-0.962) | 0.012 |  |  |
|  | American Indian/Alaska Native | 0.761 (0.490-1.180) | 0.220 | 0.842 (0.538-1.316) | 0.450 | 0.832 (0.531-1.302) | 0.420 |  |  |
| Marital status, No. (%) | |  |  |  |  |  |  |  |  |
|  | Married (including common law) | Ref. |  | Ref. |  | Ref. |  |  |  |
|  | Others | 1.520 (1.420-1.630) | <0.001 | 1.243 (1.158-1.335) | <0.001 | 1.240 (1.155-1.332) | <0.001 |  |  |
| Lateral, No. (%) | |  |  |  |  |  |  |  |  |
|  | Right colon* | Ref. |  | Ref. |  | Ref. |  |  |  |
|  | Left colon* | 1.110 (1.046-1.173) | 0.040 | 1.113 (1.034-1.198) | 0.005 | 1.111 (1.033-1.194) | 0.005 |  |  |
| Grade, No. (%) | |  |  |  |  |  |  |  |  |
|  | Well differentiated (Grade I) | Ref. |  | Ref. |  |  |  |  |  |
|  | Moderately differentiated (Grade II) | 1.410 (1.240-1.590) | <0.001 | 1.081 (0.954-1.225) | 0.220 |  |  |  |  |
|  | Poorly differentiated (Grade III) | 2.050 (1.770-2.380) | <0.001 | 1.247 (1.071-1.452) | 0.004 |  |  |  |  |
|  | Undifferentiated (Grade IV) | 1.830 (1.430-2.350) | <0.001 | 1.104 (0.860-1.418) | 0.440 |  |  |  |  |
| Histology, No. (%) | |  |  |  |  |  |  |  |  |
|  | Adenocarcinoma | Ref. |  | Ref. |  |  |  |  |  |
|  | Mucinous adenocarcinoma | 1.280 (1.140-1.440) | <0.001 | 0.987 (0.874-1.114) | 0.830 |  |  |  |  |
| T stage, No. (%) | |  |  |  |  |  |  |  |  |
|  | T1 | Ref. |  | Ref. |  | Ref. |  | Ref. |  |
|  | T2 | 1.650 (1.410-1.940) | <0.001 | 1.570 (1.336-1.844) | <0.001 | 1.594 (1.358-1.871) | <0.001 | 1.650 (1.410-1.940) | <0.001 |
|  | T3 | 3.530 (3.100-4.030) | <0.001 | 3.121 (2.715-3.588) | <0.001 | 3.239 (2.825-3.714) | <0.001 | 3.530 (3.100-4.030) | <0.001 |
|  | T4 | 9.710 (8.430-11.180) | <0.001 | 7.970 (6.809-9.329) | <0.001 | 8.688 (7.498-10.066) | <0.001 | 9.710 (8.430-11.180) | <0.001 |
| Radiotherapy, No. (%) ** | |  |  |  |  |  |  |  |  |
|  | None/Unknown | Ref. |  | Ref. |  |  |  |  |  |
|  | Yes | 2.690 (2.180-3.320) | <0.001 | 1.258 (1.007-1.572) | 0.043 |  |  |  |  |
| Chemotherapy, No. (%) | |  |  |  |  |  |  |  |  |
|  | None/Unknown | Ref. |  | Ref. |  |  |  |  |  |
|  | Yes | 1.700 (1.550-1.860) | <0.001 | 1.133 (1.012-1.267) | 0.030 |  |  |  |  |
| CEA, No. (%) | |  |  |  |  |  |  |  |  |
|  | Negative/Borderline | Ref. |  | Ref. |  | Ref. |  |  |  |
|  | Positive | 2.180 (1.990-2.390) | <0.001 | 1.515 (1.381-1.663) | <0.001 | 1.516 (1.382-1.664) | <0.001 |  |  |
|  | Unknown | 1.460 (1.350-1.590) | <0.001 | 1.364 (1.258-1.479) | <0.001 | 1.357 (1.252-1.471) | <0.001 |  |  |
| Tumor deposits, No. (%) | |  |  |  |  |  |  |  |  |
|  | No | Ref. |  | Ref. |  | Ref. |  |  |  |
|  | Yes | 3.230 (2.705-3.870) | <0.001 | 1.970 (1.645-2.361) | <0.001 | 2.021 (1.687-2.420) | <0.001 |  |  |
|  | Unknown | 1.170 (0.999-1.370) | 0.051 | 1.033 (0.872-1.225) | 0.710 | 1.033 (0.872-1.225) | 0.710 |  |  |
| Number of examined regional lymph node ≥12, No. (%) | |  |  |  |  |  |  |  |  |
|  | <12 | Ref. |  | Ref. |  | Ref. |  |  |  |
|  | ≥12 | 0.732 (0.671-0.799) | <0.001 | 0.597 (0.545-0.654) | <0.001 | 0.599 (0.547-0.656) | <0.001 |  |  |
| Perineural invasion, No. (%) | |  |  |  |  |  |  |  |  |
|  | No | Ref. |  | Ref. |  | Ref. |  |  |  |
|  | Yes | 2.260 (2.000-2.550) | <0.001 | 1.468 (1.291-1.669) | <0.001 | 1.503 (1.324-1.707) | <0.001 |  |  |
|  | Unknown | 1.240 (1.100-1.390) | <0.001 | 1.228 (1.082-1.393) | 0.002 | 1.228 (1.082-1.394) | 0.001 |  |  |

*Right colon cancer: cancer at cecum, ascending colon, hepatic flexure, and transverse colon. Left colon cancer: cancer at splenic flexure, descending colon, and sigmoid colon.

**Include beam radiation, radioactive implants, radioisotopes, or combination of these therapy.

CEA: carcinoembryonic antigen.

**Table S3. Univariate and multivariate analyses of cancer-specific death for patients in the stage III group.**

| Variables | | Univariate analysis | | Multivariate analysis | | | | | |
| --- | --- | --- | --- | --- | --- | --- | --- | --- | --- |
|  |  |  |  | Model 1 | | Model 2 | | AJCC model | |
|  |  | SHR (95% CI) | *P* | SHR (95% CI) | *P* | SHR (95% CI) | *P* | SHR (95% CI) | *P* |
| Age, No. (%) | |  |  |  |  |  |  |  |  |
|  | <65 | Ref. |  | Ref. |  | Ref. |  |  |  |
|  | ≥65 | 1.740 (1.650-1.840) | <0.001 | 1.431 (1.353-1.515) | <0.001 | 1.431 (1.352-1.515) | <0.001 |  |  |
| Sex, No. (%) | |  |  |  |  |  |  |  |  |
|  | Male | Ref. |  | Ref. |  | Ref. |  |  |  |
|  | Female | 1.050 (0.997-1.100) | 0.063 | 0.900 (0.852-0.950) | <0.001 | 0.899 (0.852-0.949) | <0.001 |  |  |
| Race, No. (%) | |  |  |  |  |  |  |  |  |
|  | White | Ref. |  | Ref. |  | Ref. |  |  |  |
|  | Black | 1.054 (0.980-1.134) | 0.150 | 1.142 (1.057-1.235) | 0.001 | 1.139 (1.054-1.231) | 0.001 |  |  |
|  | Asian or Pacific Islander | 0.904 (0.828-0.987) | 0.024 | 0.952 (0.870-1.043) | 0.290 | 0.949 (0.867-1.039) | 0.260 |  |  |
|  | American Indian/Alaska Native | 0.968 (0.727-1.289) | 0.820 | 1.053 (0.786-1.410) | 0.730 | 1.045 (0.780-1.400) | 0.770 |  |  |
| Marital status, No. (%) | |  |  |  |  |  |  |  |  |
|  | Married (including common law) | Ref. |  | Ref. |  | Ref. |  |  |  |
|  | Others | 1.380 (1.310-1.450) | <0.001 | 1.172 (1.110-1.238) | <0.001 | 1.171 (1.109-1.237) | <0.001 |  |  |
| Lateral, No. (%) | |  |  |  |  |  |  |  |  |
|  | Right colon* | Ref. |  | Ref. |  | Ref. |  |  |  |
|  | Left colon* | 0.682 (0.646-0.719) | <0.001 | 0.806 (0.760-0.854) | <0.001 | 0.801 (0.756-0.849) | <0.001 |  |  |
| Grade, No. (%) | |  |  |  |  |  |  |  |  |
|  | Well differentiated (Grade I) | Ref. |  | Ref. |  | Ref. |  |  |  |
|  | Moderately differentiated (Grade II) | 1.280 (1.110-1.460) | <0.001 | 1.179 (1.026-1.354) | 0.020 | 1.169 (1.017-1.343) | 0.027 |  |  |
|  | Poorly differentiated (Grade III) | 2.100 (1.820-2.420) | <0.001 | 1.478 (1.278-1.709) | <0.001 | 1.466 (1.268-1.695) | <0.001 |  |  |
|  | Undifferentiated (Grade IV) | 2.550 (2.160-3.020) | <0.001 | 1.644 (1.379-1.960) | <0.001 | 1.641 (1.376-1.957) | <0.001 |  |  |
| Histology, No. (%) | |  |  |  |  |  |  |  |  |
|  | Adenocarcinoma | Ref. |  | Ref. |  |  |  |  |  |
|  | Mucinous adenocarcinoma | 1.320 (1.210-1.430) | <0.001 | 1.153 (1.056-1.260) | 0.002 |  |  |  |  |
| T stage, No. (%) | |  |  |  |  |  |  |  |  |
|  | T1 | Ref. |  | Ref. |  | Ref. |  | Ref. |  |
|  | T2 | 1.740 (1.320-2.280) | <0.001 | 1.490 (1.134-1.957) | 0.004 | 1.495 (1.138-1.965) | 0.004 | 1.640 (1.250-2.160) | <0.001 |
|  | T3 | 3.740 (2.940-4.750) | <0.001 | 2.594 (2.032-3.310) | <0.001 | 2.612 (2.046-3.333) | <0.001 | 3.200 (2.510-4.070) | <0.001 |
|  | T4 | 8.090 (6.350-10.300) | <0.001 | 4.671 (3.649-5.979) | <0.001 | 4.721 (3.689-6.043) | <0.001 | 6.580 (5.160-8.390) | <0.001 |
| N stage, No. (%) | |  |  |  |  |  |  |  |  |
|  | N1 | Ref. |  | Ref. |  | Ref. |  | Ref. |  |
|  | N2 | 2.080 (1.980-2.190) | <0.001 | 1.849 (1.751-1.952) | <0.001 | 1.854 (1.756-1.958) | <0.001 | 1.830 (1.740-1.930) | <0.001 |
| Radiotherapy, No. (%) ** | |  |  |  |  |  |  |  |  |
|  | None/Unknown | Ref. |  | Ref. |  | Ref. |  |  |  |
|  | Yes | 1.460 (1.250-1.710) | <0.001 | 1.520 (1.281-1.804) | <0.001 | 1.520 (1.280-1.805) | <0.001 |  |  |
| Chemotherapy, No. (%) | |  |  |  |  |  |  |  |  |
|  | None/Unknown | Ref. |  | Ref. |  | Ref. |  |  |  |
|  | Yes | 0.496 (0.471-0.522) | <0.001 | 0.588 (0.555-0.623) | <0.001 | 0.588 (0.555-0.623) | <0.001 |  |  |
| CEA, No. (%) | |  |  |  |  |  |  |  |  |
|  | Negative/Borderline | Ref. |  | Ref. |  | Ref. |  |  |  |
|  | Positive | 1.700 (1.600-1.820) | <0.001 | 1.358 (1.270-1.452) | <0.001 | 1.361 (1.272-1.455) | <0.001 |  |  |
|  | Unknown | 1.420 (1.330-1.510) | <0.001 | 1.188 (1.114-1.268) | <0.001 | 1.189 (1.115-1.269) | <0.001 |  |  |
| Tumor deposits, No. (%) | |  |  |  |  |  |  |  |  |
|  | No | Ref. |  | Ref. |  | Ref. |  |  |  |
|  | Yes | 1.800 (1.700-1.910) | <0.001 | 1.409 (1.327-1.496) | <0.001 | 1.407 (1.325-1.494) | <0.001 |  |  |
|  | Unknown | 1.360 (1.210-1.530) | <0.001 | 1.100 (0.969-1.249) | 0.140 | 1.099 (0.968-1.248) | 0.150 |  |  |
| Number of examined regional lymph node ≥12, No. (%) | |  |  |  |  |  |  |  |  |
|  | <12 | Ref. |  | Ref. |  | Ref. |  |  |  |
|  | ≥12 | 0.742 (0.686-0.802) | <0.001 | 0.679 (0.624-0.739) | <0.001 | 0.681 (0.626-0.741) | <0.001 |  |  |
| Perineural invasion, No. (%) | |  |  |  |  |  |  |  |  |
|  | No | Ref. |  | Ref. |  | Ref. |  |  |  |
|  | Yes | 1.730 (1.630-1.840) | <0.001 | 1.331 (1.247-1.422) | <0.001 | 1.323 (1.239-1.413) | <0.001 |  |  |
|  | Unknown | 1.300 (1.190-1.420) | <0.001 | 1.158 (1.046-1.282) | 0.005 | 1.157 (1.045-1.281) | 0.005 |  |  |

*Right colon cancer: cancer at cecum, ascending colon, hepatic flexure, and transverse colon. Left colon cancer: cancer at splenic flexure, descending colon, and sigmoid colon.

**Include beam radiation, radioactive implants, radioisotopes, or combination of these therapy.

CEA: carcinoembryonic antigen.

**Table S4. Univariate and multivariate analyses of cancer-specific death for patients in the all-stage group.**

| Variables | | Univariate analysis | | Multivariate analysis | | | |
| --- | --- | --- | --- | --- | --- | --- | --- |
|  |  |  |  | Model 1 | | AJCC model | |
|  |  | SHR (95% CI) | *P* | SHR (95% CI) | *P* | SHR (95% CI) | *P* |
| Age, No. (%) | |  |  |  |  |  |  |
|  | <65 | Ref. |  | Ref. |  |  |  |
|  | ≥65 | 1.620 (1.550-1.690) | <0.001 | 1.613 (1.541-1.689) | <0.001 |  |  |
| Sex, No. (%) | |  |  |  |  |  |  |
|  | Male | Ref. |  | Ref. |  |  |  |
|  | Female | 1.080 (1.040-1.130) | <0.001 | 0.918 (0.879-0.958) | <0.001 |  |  |
| Race, No. (%) | |  |  |  |  |  |  |
|  | White | Ref. |  | Ref. |  |  |  |
|  | Black | 1.136 (1.071-1.200) | <0.001 | 1.209 (1.136-1.286) | <0.001 |  |  |
|  | Asian or Pacific Islander | 0.952 (0.884-1.020) | 0.190 | 0.912 (0.846-0.984) | 0.018 |  |  |
|  | American Indian/Alaska Native | 0.993 (0.791-1.250) | 0.950 | 1.030 (0.817-1.299) | 0.800 |  |  |
| Marital status, No. (%) | |  |  |  |  |  |  |
|  | Married (including common law) | Ref. |  | Ref. |  |  |  |
|  | Others | 1.390 (1.340-1.450) | <0.001 | 1.191 (1.140-1.243) | <0.001 |  |  |
| Lateral, No. (%) | |  |  |  |  |  |  |
|  | Right colon* | Ref. |  | Ref. |  |  |  |
|  | Left colon* | 0.851 (0.816-0.888) | <0.001 | 0.934 (0.891-0.978) | 0.004 |  |  |
| Grade, No. (%) | |  |  |  |  |  |  |
|  | Well differentiated (Grade I) | Ref. |  | Ref. |  |  |  |
|  | Moderately differentiated (Grade II) | 1.570 (1.430-1.710) | <0.001 | 1.102 (1.006-1.208) | 0.037 |  |  |
|  | Poorly differentiated (Grade III) | 3.060 (2.780-3.370) | <0.001 | 1.385 (1.253-1.531) | <0.001 |  |  |
|  | Undifferentiated (Grade IV) | 3.470 (3.060-3.940) | <0.001 | 1.492 (1.308-1.702) | <0.001 |  |  |
| Histology, No. (%) | |  |  |  |  |  |  |
|  | Adenocarcinoma | Ref. |  | Ref. |  |  |  |
|  | Mucinous adenocarcinoma | 1.360 (1.270-1.460) | <0.001 | 1.079 (1.004-1.159) | 0.038 |  |  |
| T stage, No. (%) | |  |  |  |  |  |  |
|  | T1 | Ref. |  | Ref. |  | Ref. |  |
|  | T2 | 1.840 (1.610-2.100) | <0.001 | 1.589 (1.387-1.819) | <0.001 | 1.670 (1.460-1.910) | <0.001 |
|  | T3 | 5.020 (4.480-5.610) | <0.001 | 3.238 (2.879-3.642) | <0.001 | 3.540 (3.160-3.970) | <0.001 |
|  | T4 | 12.930 (11.520-14.510) | <0.001 | 6.450 (5.696-7.305) | <0.001 | 7.760 (6.890-8.740) | <0.001 |
| N stage, No. (%) | |  |  |  |  |  |  |
|  | N0 | Ref. |  | Ref. |  | Ref. |  |
|  | N1 | 2.500 (2.380-2.620) | <0.001 | 2.002 (1.888-2.123) | <0.001 | 1.870 (1.780-1.960) | <0.001 |
|  | N2 | 5.350 (5.090-5.620) | <0.001 | 3.718 (3.485-3.967) | <0.001 | 3.480 (3.300-3.660) | <0.001 |
| Radiotherapy, No. (%) ** | |  |  |  |  |  |  |
|  | None/Unknown | Ref. |  | Ref. |  |  |  |
|  | Yes | 2.050 (1.810-2.330) | <0.001 | 1.534 (1.340-1.756) | <0.001 |  |  |
| Chemotherapy, No. (%) | |  |  |  |  |  |  |
|  | None/Unknown | Ref. |  | Ref. |  |  |  |
|  | Yes | 1.550 (1.490-1.620) | <0.001 | 0.755 (0.715-0.796) | <0.001 |  |  |
| CEA, No. (%) | |  |  |  |  |  |  |
|  | Negative/Borderline | Ref. |  | Ref. |  |  |  |
|  | Positive | 2.060 (1.960-2.180) | <0.001 | 1.411 (1.337-1.490) | <0.001 |  |  |
|  | Unknown | 1.380 (1.320-1.450) | <0.001 | 1.265 (1.203-1.329) | <0.001 |  |  |
| Tumor deposits, No. (%) | |  |  |  |  |  |  |
|  | No | Ref. |  | Ref. |  |  |  |
|  | Yes | 3.260 (3.100-3.430) | <0.001 | 1.438 (1.358-1.523) | <0.001 |  |  |
|  | Unknown | 1.510 (1.380-1.650) | <0.001 | 1.168 (1.059-1.288) | 0.002 |  |  |
| Number of examined regional lymph node ≥12, No. (%) | |  |  |  |  |  |  |
|  | <12 | Ref. |  | Ref. |  |  |  |
|  | ≥12 | 0.830 (0.783-0.879) | <0.001 | 0.639 (0.600-0.680) | <0.001 |  |  |
| Perineural invasion, No. (%) | |  |  |  |  |  |  |
|  | No | Ref. |  | Ref. |  |  |  |
|  | Yes | 2.670 (2.530-2.810) | <0.001 | 1.329 (1.253-1.410) | <0.001 |  |  |
|  | Unknown | 1.390 (1.290-1.490) | <0.001 | 1.211 (1.119-1.311) | <0.001 |  |  |

*Right colon cancer: cancer at cecum, ascending colon, hepatic flexure, and transverse colon. Left colon cancer: cancer at splenic flexure, descending colon, and sigmoid colon.

**Include beam radiation, radioactive implants, radioisotopes, or combination of these therapy.

CEA: carcinoembryonic antigen.

**Table S5. Multivariate analysis of cancer-specific death for patients in the Xi’an cohort.**

| Variables | | Multivariate analysis | | | | | |
| --- | --- | --- | --- | --- | --- | --- | --- |
|  |  | Stage I/II | | Stage III | | All stage | |
|  |  | SHR (95% CI) | *P* | SHR (95% CI) | *P* | SHR (95% CI) | *P* |
| Age, No. (%) | |  |  |  |  |  |  |
|  | <65 | Ref. |  | Ref. |  | Ref. |  |
|  | ≥65 | 1.563 (1.082-2.257) | 0.017 | 1.171 (0.759-1.806) | 0.480 | 1.408 (1.065-1.862) | 0.016 |
| Sex, No. (%) | |  |  |  |  |  |  |
|  | Male | Ref. |  | Ref. |  | Ref. |  |
|  | Female | 0.903 (0.617-1.321) | 0.600 | 0.849 (0.553-1.301) | 0.450 | 0.909 (0.688-1.202) | 0.500 |
| Marital status, No. (%) | |  |  |  |  |  |  |
|  | Married (including common law) | Ref. |  | Ref. |  | Ref. |  |
|  | Others | 1.632 (1.129-2.360) | 0.009 | 1.736 (1.133-2.661) | 0.011 | 1.677 (1.275-2.207) | <0.001 |
| Lateral, No. (%) | |  |  |  |  |  |  |
|  | Right colon* | Ref. |  | Ref. |  | Ref. |  |
|  | Left colon* | 1.239 (0.838-1.832) | 0.280 | 0.854 (0.547-1.332) | 0.490 | 1.090 (0.820-1.449) | 0.550 |
| Grade, No. (%) | |  |  |  |  |  |  |
|  | Well differentiated (Grade I) | Ref. |  | Ref. |  | Ref. |  |
|  | Moderately differentiated (Grade II) | 0.908 (0.392-2.102) | 0.820 | 1.188 (0.310-4.558) | 0.800 | 1.030 (0.505-2.099) | 0.940 |
|  | Poorly differentiated (Grade III) | 1.338 (0.500-3.579) | 0.560 | 2.548 (0.648-10.017) | 0.180 | 1.964 (0.912-4.229) | 0.084 |
| Histology, No. (%) | |  |  |  |  |  |  |
|  | Adenocarcinoma | Ref. |  | Ref. |  | Ref. |  |
|  | Mucinous adenocarcinoma | 2.182 (0.472-10.085) | 0.320 | 3.412 (0.553-21.053) | 0.190 | 2.531 (0.942-6.802) | 0.066 |
| T stage, No. (%) | |  |  |  |  |  |  |
|  | T1/2 | Ref. |  | Ref. |  | Ref. |  |
|  | T3/4 | 3.841 (1.578-9.349) | 0.003 | 2.070 (1.004-4.268) | 0.049 | 2.544 (1.465-4.420) | <0.001 |
| N stage, No. (%) | |  |  |  |  |  |  |
|  | N0 | - |  | - |  | Ref. |  |
|  | N1 | - |  | Ref. |  | 1.028 (0.737-1.433) | 0.870 |
|  | N2 | - |  | 2.023 (1.357-3.016) | <0.001 | 2.018 (1.413-2.880) | <0.001 |
| Radiotherapy, No. (%) ** | |  |  |  |  |  |  |
|  | None/Unknown | Ref. |  | Ref. |  | Ref. |  |
|  | Yes | 0.821 (0.431-1.567) | 0.550 | 0.647 (0.284-1.473) | 0.300 | 0.735 (0.436-1.239) | 0.250 |
| Chemotherapy, No. (%) | |  |  |  |  |  |  |
|  | None/Unknown | Ref. |  | Ref. |  | Ref. |  |
|  | Yes | 0.304 (0.172-0.536) | <0.001 | 0.498 (0.288-0.863) | 0.013 | 0.390 (0.265-0.574) | <0.001 |
| CEA, No. (%) | |  |  |  |  |  |  |
|  | Negative/Borderline | Ref. |  | Ref. |  | Ref. |  |
|  | Positive | 0.898 (0.562-1.436) | 0.650 | 1.933 (1.122-3.331) | 0.018 | 1.238 (0.881-1.738) | 0.220 |
|  | Unknown | 1.006 (0.649-1.559) | 0.980 | 1.734 (0.989-3.039) | 0.055 | 1.258 (0.900-1.757) | 0.180 |
| Tumor deposits, No. (%) | |  |  |  |  |  |  |
|  | No | Ref. |  | Ref. |  | Ref. |  |
|  | Yes | 1.791 (1.121-2.862) | 0.015 | 1.530 (0.913-2.565) | 0.110 | 1.620 (1.160-2.263) | 0.005 |
| Number of examined regional lymph node ≥12, No. (%) | |  |  |  |  |  |  |
|  | <12 | Ref. |  | Ref. |  | Ref. |  |
|  | ≥12 | 0.738 (0.463-1.178) | 0.200 | 0.885 (0.521-1.501) | 0.650 | 0.826 (0.588-1.160) | 0.270 |
| Perineural invasion, No. (%) | |  |  |  |  |  |  |
|  | No | Ref. |  | Ref. |  | Ref. |  |
|  | Yes | 0.991 (0.638-1.541) | 0.970 | 1.747 (1.099-2.778) | 0.018 | 1.314 (0.953-1.811) | 0.095 |

*Right colon cancer: cancer at cecum, ascending colon, hepatic flexure, and transverse colon. Left colon cancer: cancer at splenic flexure, descending colon, and sigmoid colon.

**Include beam radiation, radioactive implants, radioisotopes, or combination of these therapy.

CEA: carcinoembryonic antigen.

**Table S6. The proportional hazard assumption test for models.**

|  | Stage I/II | | | Stage III | | | All stage | | |
| --- | --- | --- | --- | --- | --- | --- | --- | --- | --- |
|  | Model 1 | Model 2 | AJCC  model | Model 1 | Model 2 | AJCC  model | | Model 1 | AJCC  model |
| Age, No. (%) | <0.001 | <0.001 | NA | <0.001 | <0.001 | NA | | <0.001 | NA |
| Marital status, No. (%) | <0.001 | <0.001 | NA | <0.001 | <0.001 | NA | | <0.001 | NA |
| Sex, No. (%) | 0.002 | 0.002 | NA | <0.001 | <0.001 | NA | | <0.001 | NA |
| Race, No. (%) | 0.003 | 0.003 | NA | <0.001 | <0.001 | NA | | <0.001 | NA |
| Lateral, No. (%) | <0.001 | <0.001 | NA | <0.001 | <0.001 | NA | | <0.001 | NA |
| Grade, No. (%) | <0.001 | NA | NA | <0.001 | <0.001 | NA | | <0.001 | NA |
| Histology, No. (%) | 0.45 | NA | NA | 0.024 | NA | NA | | 0.085 | NA |
| T stage, No. (%) | 0.147 | 0.15 | 0.055 | <0.001 | <0.001 | <0.001 | | 0.036 | <0.001 |
| N stage, No. (%) | NA | NA | NA | <0.001 | <0.001 | <0.001 | | 0.48 | 0.001 |
| Radiotherapy, No. (%) | <0.001 | NA | NA | 0.007 | 0.007 | NA | | <0.001 | NA |
| Chemotherapy, No. (%) | <0.001 | NA | NA | <0.001 | <0.001 | NA | | <0.001 | NA |
| CEA, No. (%) | <0.001 | <0.001 | NA | <0.001 | <0.001 | NA | | <0.001 | NA |
| Tumor deposits, No. (%) | 0.063 | 0.061 | NA | 0.03 | 0.03 | NA | | 0.058 | NA |
| Number of examined regional lymph node ≥12, No. (%) | 0.104 | 0.097 | NA | 0.096 | 0.094 | NA | | <0.001 | NA |
| Perineural invasion, No. (%) | 0.671 | 0.695 | NA | 0.282 | 0.282 | NA | | 0.706 | NA |
| GLOBAL | <0.001 | <0.001 | 0.055 | <0.001 | <0.001 | <0.001 | | <0.001 | <0.001 |

CEA: carcinoembryonic antigen.
